# Early vs. Delayed Initiation of Treatment With P2Y_12_ Inhibitors in Patients With Non-ST-Segment Elevation Acute Coronary Syndrome: A Systematic Review and Network Meta-Analysis of Randomized Controlled Trials

**DOI:** 10.3389/fcvm.2022.862452

**Published:** 2022-04-28

**Authors:** Lourdes Vicent, Carlos Diaz-Arocutipa, Giuseppe Tarantini, Marco Mojoli, Adrian V. Hernandez, Héctor Bueno

**Affiliations:** ^1^Cardiology Department, Hospital Universitario 12 de Octubre and Instituto de Investigación Sanitaria Hospital 12 de Octubre (imas12), Madrid, Spain; ^2^Centro de Investigación Biomédica en Red Enfermedades Cardiovasculares (CIBERCV), Madrid, Spain; ^3^Vicerrectorado de Investigación, Universidad San Ignacio de Loyola, Lima, Peru; ^4^Department of Cardiac, Thoracic and Vascular Sciences, University of Padua Medical School, Padua, Italy; ^5^Cardiology Department, Azienda Ospedaliera Friuli Occidentale, Pordenone, Italy; ^6^Health Outcomes, Policy, and Evidence Synthesis (HOPES) Group, University of Connecticut School of Pharmacy, Storrs, CT, United States; ^7^Centro Nacional de Investigaciones Cardiovasculares (CNIC), Madrid, Spain; ^8^Facultad de Medicina, Universidad Complutense de Madrid, Madrid, Spain

**Keywords:** clopidogrel, prasugrel, ticagrelor, P2Y_12_ inhibitors, Non-ST-segment elevation acute coronary syndrome, network meta-analysis

## Abstract

**Aims:**

Whether early or delayed dual antiplatelet therapy initiation is better in patients with non-ST-segment elevation acute coronary syndrome (NSTE-ACS) is unclear. We assessed the evidence for comparing the efficacy and safety of early vs. delayed P2Y_12_ inhibitor initiation in NSTE-ACS.

**Methods:**

The randomized controlled trials with available comparisons between early and delayed initiation of P2Y_12_ inhibitors (clopidogrel, prasugrel, and ticagrelor) in patients with NSTE-ACS until January 2021 were reviewed. The primary outcomes were trial-defined major adverse cardiovascular events (MACEs) and bleeding. Secondary outcomes were all-cause mortality, cardiovascular mortality, myocardial infarction, stent thrombosis, urgent coronary revascularization, and stroke. Frequentist random-effects network meta-analyses were conducted, ranking best treatments per outcome with *p*-scores.

**Results:**

A total of nine trials with intervention arms including early and delayed initiation of clopidogrel (*n* = 5), prasugrel (*n* = 8), or ticagrelor (*n* = 6) involving 40,096 patients were included. Early prasugrel (hazard ratio [HR], 0.59; 95% confidence interval [95%CI], 0.40–0.87), delayed prasugrel (HR, 0.60; 95%CI 0.43–0.84), and early ticagrelor (HR, 0.84; 95%CI, 0.74–0.96) significantly reduced MACE compared with early clopidogrel, but increased bleeding risk. Delayed prasugrel ranked as the best treatment to reduce MACE (*p*-score=0.80), early prasugrel to reduce all-cause mortality, cardiovascular mortality, stent thrombosis, and stroke, and delayed clopidogrel to reduce bleeding (*p*-score = 0.84). The risk of bias was low for all trials.

**Conclusion:**

In patients with NSTE-ACS, delayed prasugrel initiation was the most effective strategy to reduce MACE. Although early prasugrel was the best option to reduce most secondary cardiovascular outcomes, it was associated with the highest bleeding risk. The opposite was found for delayed clopidogrel.

## Background

Dual antiplatelet therapy (DAPT) including aspirin and a P2Y_12_ inhibitor is a cornerstone in the treatment of patients with acute coronary syndrome (ACS) (1–3). This strong platelet inhibition reduces the thrombotic burden, improving outcomes but increasing the risk of bleeding with differences between antiplatelets, which must be balanced for drug selection. The optimal timing for the initiation of P2Y_12_ inhibitors in patients with non-ST-segment elevation ACS (NSTE-ACS) is controversial (2, 4, 5). While early inhibition—that is, immediately after a clinical diagnosis of ACS is established— may prevent the progression of coronary thrombosis, reducing the risk of further myocardial ischemic events and improving the results of coronary intervention (4–6), it may increase bleedings (4, 6). A delayed strategy of P2Y_12_ inhibitor initiation —most often, started when coronary anatomy is known by coronary angiography and a strategy of percutaneous coronary intervention (PCI) has been decided— should decrease bleeding risk but may reduce as well the potential benefits of early antithrombotic treatment. The latest European Society of Cardiology (ESC) guidelines on NSTE-ACS (2) changed their recommendation about the timing of P2Y_12_ inhibitor administration compared with the previous guidelines (7, 8) and recommended to avoid an early treatment as a routine strategy in patients in whom coronary anatomy is unknown when early invasive management is planned (2). This recommendation is mainly based on the two randomized clinical trials (RCT) (6, 9) one of them not specifically designed to evaluate the role of early treatment in the treatment of patients with NSTE-ACS (9). Actually, the scarce information on the best timing for initiating DAPT in patents with NSTE-ACS is a limitation as the number of studies specifically designed to address the potential benefit or harm of an early vs. a delayed administration of P2Y_12_ inhibitors is reduced (6, 10). We performed a systematic review and network meta-analysis of all RCTs in which comparisons between early and delayed initiation of P2Y_12_ inhibitors in patients with NSTE-ACS were available to estimate the potential differences in benefits and risks between the early and delayed initiation strategies with different P2Y_12_ inhibitors.

## Methods

This review was reported according to the PRISMA for Network Meta-Analyses (Preferred Reporting Items for Systematic Reviews and Meta-Analyses) statement (11) and registered in the PROSPERO database (registration ID: CRD42021268026).

### Search Strategy

PubMed, EMBASE, Scopus, Web of Science, and CENTRAL were searched from each database inception to 9 January 2021. The complete search strategy is available in Supplementary Table S1. There were no language restrictions. We also performed hand searches of reference lists of included RCTs and relevant to identify other potentially eligible studies.

### Eligibility Criteria

Study inclusion criteria were as follows: RCTs (i) comparing the results of at least one P2Y_12_ inhibitor treatment (clopidogrel, prasugrel, or ticagrelor) started before coronary angiography (early treatment) and after coronary angiography (delayed treatment) in patients with NSTE-ACS, (ii) enrolling adult patients (≥18 years old), and (iii) reporting at least one of the primary or secondary outcomes at any length of follow-up. Observational studies, case series, case reports, systematic reviews, conference abstracts, and editorials were excluded.

### Study Selection

All articles from the electronic search were downloaded into EndNote X8 and duplicate records were removed. All unique articles were uploaded to Rayyan (https://rayyan.qcri.org/) for the study selection process. Titles and abstracts were independently screened by the two investigators (LV and CDA) to identify the relevant studies. The same investigators independently examined full texts of selected studies and registered reasons for exclusions. Disagreements were resolved by consensus.

### Outcomes

The primary outcomes were major adverse cardiovascular events (MACEs) and bleeding. Secondary outcomes were all-cause mortality, cardiovascular mortality, myocardial infarction, stent thrombosis, urgent coronary revascularization, and stroke. Trial definitions were used for all outcomes (Supplementary Table S2).

### Data Extraction

The two investigators (LV and CDA) independently extracted the data using a standardized data extraction form that was previously piloted. Disagreements were resolved by a third investigator (AVH). If additional data were needed, we contacted the corresponding author by email to request further information. We extracted the following information: first author name, year of publication, country, study design, population, sample size, age, sex, description of intervention arms, follow-up duration, and primary and secondary outcomes per strategy arm.

### Risk of Bias Assessment

Supplementary Figure S1 summarizes the risk of bias of included studies. The two investigators (LV and CDA) independently assessed the risk of bias for each RCT using the Cochrane Risk of Bias (RoB) tool 2.0 (11). Disagreements were resolved by a third investigator (AVH). The RoB 2.0 tool evaluates five domains: randomization process, deviations from intended interventions, missing outcome data, measurement of the outcome, and selection de the reported result. Each domain per RCT and each RCT overall was judged as having low, some concerns, or high risk of bias.

### Statistical Analyses

To compare therapeutic time strategies with P2Y_12_ inhibitors (early clopidogrel, delayed clopidogrel, early prasugrel, delayed prasugrel, early ticagrelor, and delayed ticagrelor), we performed network meta-analyses within a frequentist framework. Inverse variance random-effects models were used. Effects of treatment strategies on dichotomous outcomes were expressed as relative risks (RRs) or hazard ratios (HRs) with their 95% confidence intervals (CIs). For the primary outcomes, the main analyses were performed using HRs and, as secondary analyses, RRs were used. For all secondary outcomes, RRs were pooled.

The transitivity assumption was assessed by comparing patient and trial characteristics (type of NSTE-ACS, timing and dosage of P2Y_12_ inhibitors, revascularization strategy, and outcomes) across RCTs. Consistency between direct and indirect effects was evaluated using the design-by-treatment interaction test for the overall network (12). Heterogeneity was assessed using the I^2^ statistic and defined as low if I^2^ <30%, moderate if I^2^ = 30–60%, and high if I^2^ > 60%. The ranking among treatments per outcome (i.e., best to worst) was calculated using the *p*-scores (13). Publication bias was not evaluated since the number of RCTs per outcome was less than 10.

Subgroups analyses were performed according to the type of NSTE-ACS [unstable angina vs. non-ST elevation myocardial infarction (NSTEMI)] and type of revascularization strategy (PCI vs. coronary artery bypass grafting vs. medical therapy) if enough number of RCTs was available. We also conducted sensitivity analyses as follows: (i) excluding one RCT from the main analyses as patients received only medical therapy (14) and (ii) including only the delayed treatment groups of the Tarantini et al.'s RCT (10). We used the packages *meta* and *netmeta* from R 3.6.3 (www.r-project.org) for all meta-analyses. A two-tailed *p* < 0.05 was considered statistically significant.

## Results

### Study Selection

**Figure 1:**
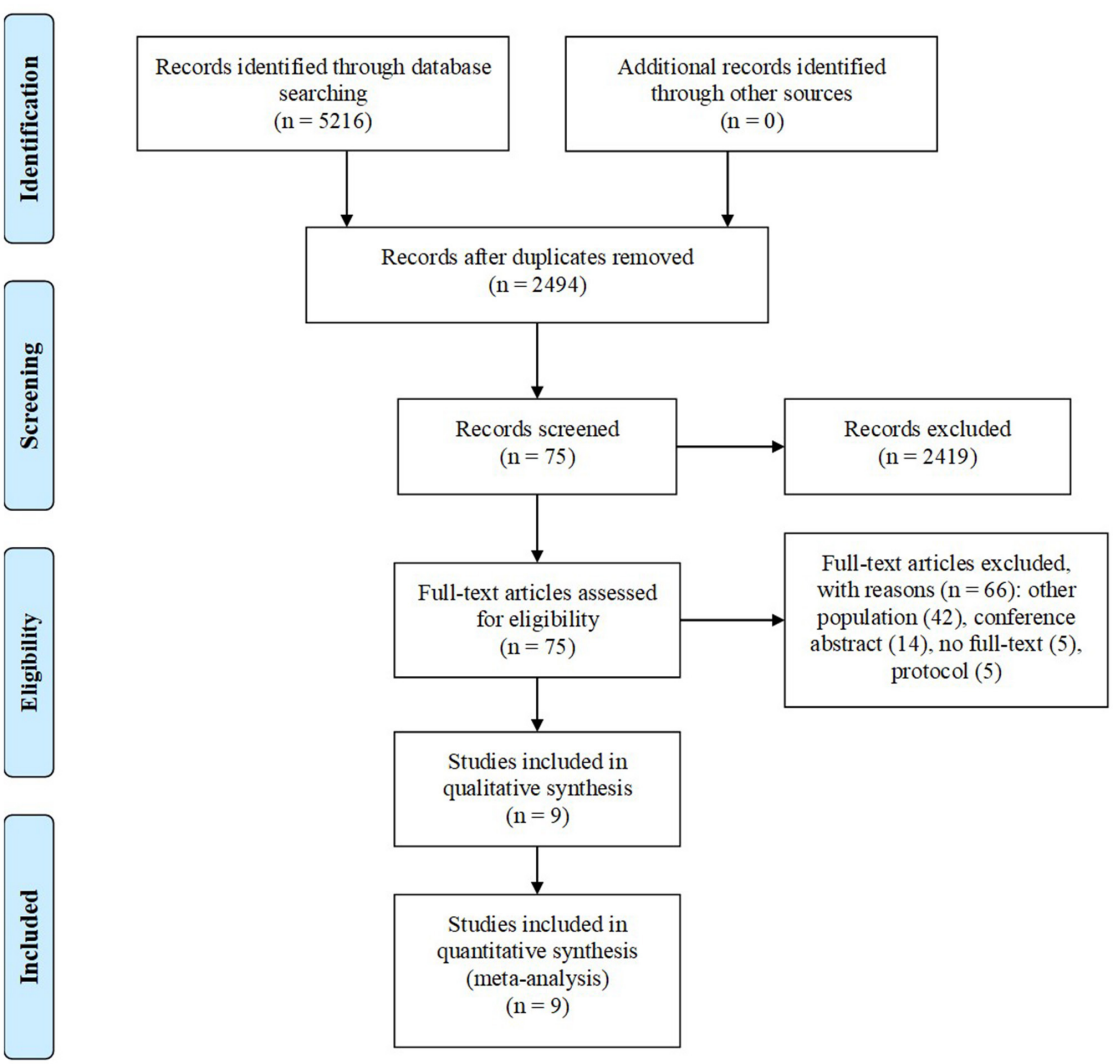
Diagram flow of study selection.

Our search strategy identified 5,216 unique articles. After the removal of duplicates, 2,494 articles remained. After screening of articles by title or abstract, 2,419 articles were excluded. After full-text assessment of 75 articles, 66 articles were excluded for the following reasons: other population (*n* = 42), conference abstract (*n* = 14), no full-text (*n* = 5), and protocol (*n* = 5). A total of nine RCTs were finally selected (Figure 1) (6, 10, 14–20).

### Trial Characteristics

The main characteristics of the selected RCTs are summarized in Table 1. A total of 40,096 patients were included, with sample sizes ranging from 213 to 11,080 patients. The mean ages ranged between 60 and 66 years and 71% were men (Supplementary Table S3). A total of five out of nine RCTs were conducted in several countries. Follow-up time ranged from one to 17.1 months. The proportion of patients with NSTEMI ranged from 46 to 100% (Supplementary Table S3). The use of PCI ranged from 46 to 100% across RCTs.

**Table 1:** Characteristics of included randomized controlled trials.

**Acronym & Author, Year [reference]**	**Country (ies)**	**Type of RCT**	**Sample size**	**Population**	**Follow-up time (months)**	**Arms**	**Timing**	**Revascularization strategy**	**Outcomes**
TRILOGY-ACS Roe, 2012 (14)	Various countries	Parallel, double-blinded	9,326	NSTE-ACS patients with medical treatment without revascularization within 10 days after the index event	17.1 (10.4-24.4)	Prasugrel	DT	Medical treatment (100%)	MACE, CV mortality, MI, stroke, bleeding
						Clopidogrel	DT	Medical treatment (100%)	
ACCOAST Montalescot, 2013 (6)	Various countries	Parallel, double-blinded	4,033	NSTEMI who underwent PCI 2-48 h after randomization	1	Prasugrel	ET	PCI (68.7%), CABG (6.2%), medical treatment (25.1%)	MACE, all-cause mortality, CV mortality, MI, stroke, urgent coronary revascularization, stent thrombosis, bleeding
						Prasugrel	DT		
TRITON-TIMI 38 De Servi, 2014 (15)	Various countries	Parallel, double-blinded	10,074	Moderate to high risk NSTE-ACS with scheduled PCI	14.5 (6-15)	Prasugrel	DT	PCI (99.1%)	MACE, CV mortality, MI, stroke, bleeding
						Clopidogrel	DT	PCI (99.1%)	
PLATO Lindholm, 2014 (16)	Various countries	Parallel, double-blinded	11,080	Patients with NSTE-ACS	12	Ticagrelor	ET	PCI (51.4%), CABG (12.1%), medical treatment (36.5%)	MACE, all-cause mortality, CV mortality, MI, stroke, bleeding
						Clopidogrel	ET	PCI (51.7%), CABG (12.3%), medical treatment (36%)	
Bonello, 2015 (17)	France	Parallel, open-label	213	Adult patients who underwent PCI for an intermediate or high-risk NSTE-ACS	1	Ticagrelor	ET	PCI (100%)	MACE, CV mortality, MI, stroke, bleeding
						Prasugrel	DT	PCI (100%)	
Elderly ACS 2 Savonitto, 2018 (18)	Italy	Parallel, open-label	848	Patients >74 years with ACS treated with PCI during index admission	12	Prasugrel	DT	PCI (99.8%)	MACE, stent thrombosis
						Clopidogrel	DT		
POPULAR AGE Gimbel, 2020 (19)	Netherlands	Parallel, open-label	1,002	Patients with NSTE-ACS aged 70 years or older randomized within 72 hrs after admission	12	Ticagrelor	ET	PCI (48%), CABG (17%), medical treatment (35%)	MACE, all-cause mortality, CV mortality, MI, stroke, urgent coronary revascularization, stent thrombosis, bleeding
						Clopidogrel	ET	PCI (46%), CABG (16%), medical treatment (38%)	
ISAR-REACT-5 Valina, 2020 (20)	Germany and Italy	Parallel, open-label	2,365	Patients with NSTE-ACS scheduled to coronary angiography	12	Ticagrelor	ET	PCI (76.3%), CABG (3.59%), medical treatment (20.2%)	MACE, all-cause mortality, CV mortality, MI, stroke, stent thrombosis, bleeding
						Prasugrel	DT	PCI (77.0%), CABG (2.78%), medical treatment (20.3%)	
DUBIUS Tarantini, 2020 (10)	Italy	Parallel, open-label	1,155	Patients with NSTE-ACS scheduled to coronary angiography within 72hrs from hospital admission	1	Ticagrelor	ET	PCI (70.1%), CABG (6.6%), medical treatment (23.2%)	MACE, all-cause mortality, CV mortality, MI, stroke, urgent coronary revascularization, stent thrombosis, bleeding
						Prasugrel	DT	PCI (96.8%), CABG (0%), medical treatment (3.2%)	
						Ticagrelor	DT	PCI (97.7%), CABG (0.5%), medical treatment (1.8%)	

*RCT, randomized controlled trial; NSTE-ACS, non-ST-segment elevation acute coronary syndrome; PCI, percutaneous coronary intervention; MACE, major adverse cardiovascular events; CV, cardiovascular; MI, myocardial infarction; CABG, coronary artery bypass grafting; ET: early treatment; DT: delayed treatment*.

Early treatment with clopidogrel was assessed in two studies, delayed treatment with clopidogrel in three studies, early treatment with prasugrel in one study, delayed treatment with prasugrel in seven studies, early treatment with ticagrelor in five studies, and delayed treatment with ticagrelor in one study (specific data not published, provided by the authors of the RCT (10)). The dosages of P2Y_12_ inhibitors were as follows: clopidogrel 300–600 mg as loading dose, then 75 mg one time a day; prasugrel 30–60 mg as loading dose, then 5–10 mg one time a day; and ticagrelor 180 mg loading dose, then 90 mg two times a day (Supplementary Table S3).

Network geometries for MACE, bleeding, all-cause mortality, cardiovascular mortality, myocardial infarction, stent thrombosis, and stroke showed direct comparisons for early treatment with clopidogrel vs. early treatment with ticagrelor, delayed treatment with clopidogrel vs. delayed treatment with prasugrel, early treatment with ticagrelor vs. delayed treatment with prasugrel, early treatment with ticagrelor vs. delayed treatment with ticagrelor, delayed treatment with prasugrel vs. delayed treatment with ticagrelor, and delayed treatment with prasugrel vs. early treatment with prasugrel. For urgent coronary revascularization, the geometry of the network showed the same direct comparisons as the other outcomes, except for delayed treatment with clopidogrel (Supplementary Figure S2).

### Risk of Bias Assessment

All RCTs were evaluated as of low risk of bias for all domains.

### Network Meta-Analyses of Primary Outcomes

The effects of P2Y_12_ inhibitors on primary and secondary outcomes using early treatment with clopidogrel as control group in network meta-analyses are described in Supplementary Figure S3. Direct and indirect results for all comparisons among treatment arms are shown in Supplementary Figures S3–S12.

#### Mace

Using HR as effect measure, delayed treatment with prasugrel (HR 0.60; 95% CI 0.43–0.84), early treatment with prasugrel (HR 0.59; 95% CI 0.40–0.87), and early treatment with ticagrelor (HR 0.84; 95% CI 0.74–0.96) had a significant reduction of MACE compared with early treatment with clopidogrel (Table 2). In addition, early treatment with ticagrelor showed a significant increase of MACE compared with delayed treatment with prasugrel (HR 1.40; 95% CI 1.03–1.91) (Table 2). For these analyses, the six treatment strategies were available (early/delayed administration of clopidogrel, prasugrel, and ticagrelor). Heterogeneity of effects was low (I^2^ = 14%) and the overall inconsistency was not significant (*p* = 0.36).

**Table 2:** League table of the effects of P2Y_12_ inhibitors expressed as hazard ratio with their 95% CIs on MACE (white cells) and bleeding (gray cells).

**Delayed clopidogrel**	**0.61 (0.30–1.23)**	**0.76 (0.53–1.08)**	**0.38 (0.19–0.77)**	**1.08 (0.32–3.69)**	**0.68 (0.36–1.27)**
**1.50 (1.06–2.12)**	**Early clopidogrel**	**1.24 (0.68–2.27)**	**0.63 (0.27–1.47)**	**1.77 (0.53–5.97)**	**1.11 (0.82–1.51)**
**0.90 (0.82–0.99)**	**0.60 (0.43–0.84)**	**Delayed prasugrel**	**0.51 (0.28–0.92)**	**1.43 (0.44–4.62)**	**0.89 (0.53–1.50)**
**0.89 (0.72–1.10)**	**0.59 (0.40–0.87)**	**0.99 (0.82–1.20)**	**Early prasugrel**	**2.81 (0.75–10.47)**	**1.76 (0.80–3.86)**
**0.98 (0.36–2.68)**	**0.65 (0.24–1.78)**	**1.09 (0.40–2.98)**	**1.10 (0.39–3.06)**	**Delayed ticagrelor**	**0.63 (0.19–2.02)**
**1.26 (0.91–1.74)**	**0.84 (0.74–0.96)**	**1.40 (1.03–1.91)**	**1.42 (0.98–2.04)**	**1.29 (0.48–3.49)**	**Early ticagrelor**

*CIs, confidence intervals; MACE, major adverse cardiovascular events. For hazard ratios of MACE and bleeding the comparison is row vs. column (comparator). Effects in bold are statistically significant*.

Using RR as effect measure, only delayed treatment with prasugrel had a significant reduction of MACE compared with early treatment with clopidogrel (RR, 0.68; 95% CI, 0.47–0.97) (Table 3). For these analyses, the six treatment strategies were available. The heterogeneity of effects was moderate (I^2^ = 41%) and the overall inconsistency was not significant (*p* = 0.37).

**Table 3:** League table of the effects of P2Y_12_ inhibitors expressed as risk ratio with their 95% CIs on MACE (white cells) and bleeding (gray cells).

**Delayed clopidogrel**	**0.78 (0.50–1.22)**	**0.76 (0.59–0.96)**	**0.39 (0.23–0.65)**	**0.88 (0.29–2.69)**	**0.70 (0.45–1.08)**
**1.32 (0.90–1.93)**	**Early clopidogrel**	**0.97 (0.66–1.42)**	**0.49 (0.27–0.89)**	**1.12 (0.38–3.31)**	**0.90 (0.79–1.02)**
**0.89 (0.78–1.03)**	**0.68 (0.47–0.97)**	**Delayed prasugrel**	**0.51 (0.32–0.80)**	**1.16 (0.39–3.47)**	**0.93 (0.65–1.33)**
**0.89 (0.67–1.17)**	**0.67 (0.44–1.04)**	**0.99 (0.78–1.26)**	**Early prasugrel**	**2.27 (0.69–7.44)**	**1.82 (1.02–3.24)**
**0.32 (0.04–2.48)**	**0.24 (0.03–1.87)**	**0.36 (0.05–2.77)**	**0.36 (0.05–2.83)**	**Delayed ticagrelor**	**0.80 (0.27–2.34)**
**1.15 (0.82–1.62)**	**0.88 (0.73–1.05)**	**1.29 (0.95–1.76)**	**1.30 (0.88–1.93)**	**3.59 (0.47–27.34)**	**Early ticagrelor**

*CIs, confidence intervals; MACE, major adverse cardiovascular events. For risk ratios of MACE and bleeding the comparison is row vs. column (comparator). Effects in bold are statistically significant*.

**Figure 2:**
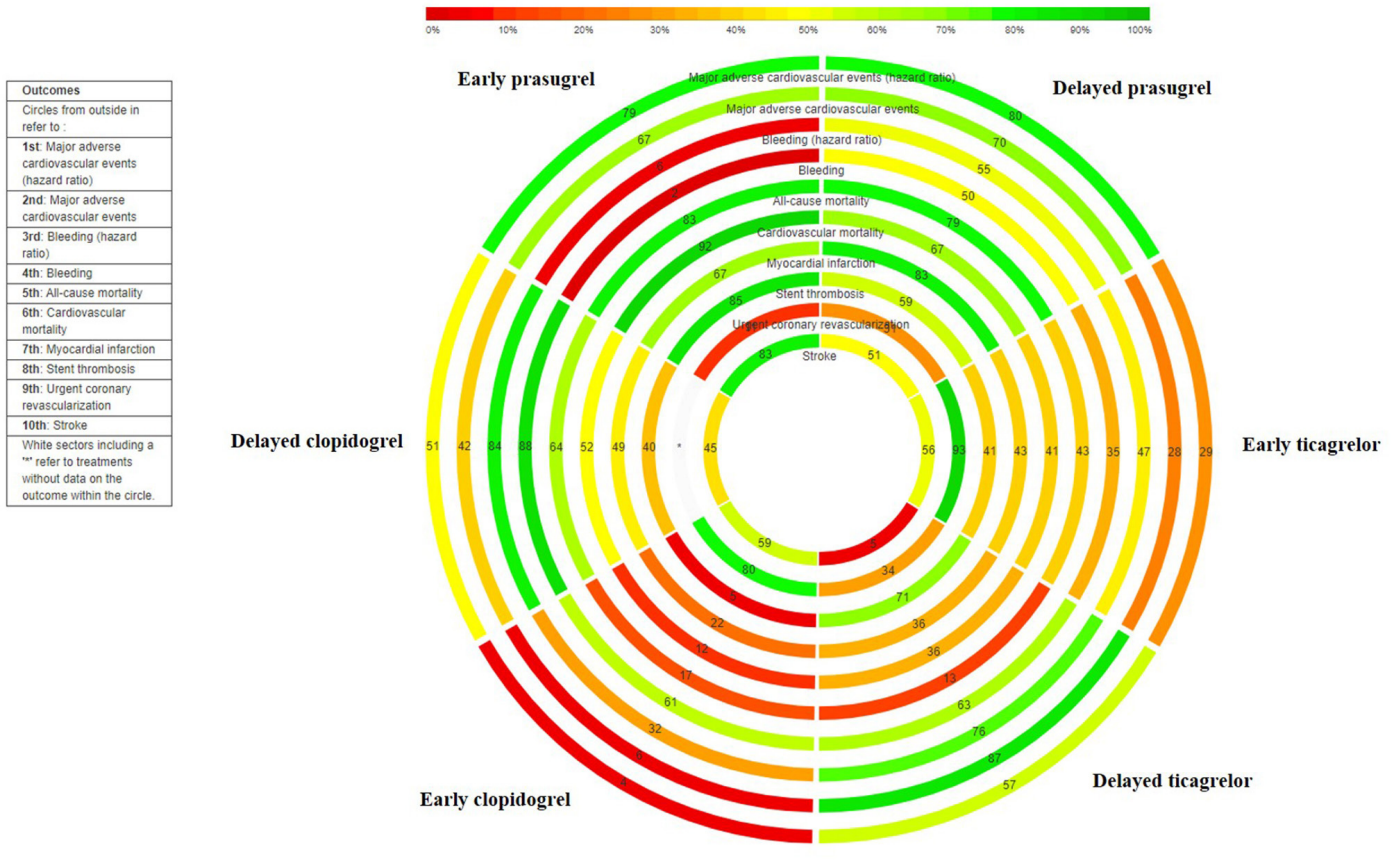
Rank-heat plot. Each concentric circle represents a different outcome (as labeled), with the outermost circle representing the MACEs, and the innermost circle representing stroke. The scale bar represents the ranking statistic for each intervention using the *p*-scores, where 0% (red) indicates the lowest possible rank (worst treatment), and 100% (green) represents the highest possible rank (best treatment). Each rectangle represents an intervention and is coded using a letter outside the outmost circle (see treatment legend). The number within each rectangle represents the ranking statistic of the intervention for the particular outcome circle.

Delayed treatment with prasugrel ranked as the best intervention for reducing MACE among all the treatments (*p*-score = 0.80) and early treatment with clopidogrel ranked as the worst intervention (*p*-score = 0.04) (Figure 2 and Supplementary Table S4).

#### Bleeding

Using HR as effect measure, delayed treatment with clopidogrel (HR, 0.38; 95% CI, 0.19–0.77) and delayed treatment with prasugrel (HR, 0.51; 95% CI, 0.28–0.92) showed a significant reduction of bleeding risk compared to early treatment with prasugrel (Table 2). For these analyses, the six treatment strategies were available. Heterogeneity of effects was high (I^2^ = 62%) and the overall inconsistency was not significant (*p* = 0.67).

Using RR as effect measure, delayed treatment with clopidogrel (RR 0.39; 95% CI 0.23–0.65), early treatment with clopidogrel (RR 0.49; 95% CI 0.27–0.89), and delayed treatment with prasugrel (RR 0.51; 95% CI 0.32–0.80) showed a significant reduction of bleeding risk compared with early treatment with prasugrel (Table 3). Delayed treatment with clopidogrel showed a significant reduction of bleeding risk compared with delayed treatment with prasugrel (RR, 0.76; 95% CI, 0.59–0.96). Early treatment with prasugrel showed a significant increase of bleeding risk compared with early treatment with ticagrelor (RR, 1.82; 95% CI, 1.02–3.24). For these analyses, all six treatment strategies were available. Heterogeneity of effects was low (I^2^ = 10%) and the overall inconsistency was not significant (*p* = 0.41).

Delayed treatment with clopidogrel ranked as the best intervention for reducing bleeding among all the treatments (*p*-score = 0.84) and early treatment with prasugrel ranked as the worst intervention (*p*-score = 0.06) (Figure 2 and Supplementary Table S4).

### Network Meta-Analyses of Secondary Outcomes

#### All-Cause Mortality

Early treatment with clopidogrel was associated with a significant increase in all-cause mortality risk compared with delayed treatment with clopidogrel (RR 1.69; 95% CI 1.06–2.70) (Supplementary Table S4). Delayed treatment with prasugrel (RR 0.56; 95% CI 0.35–0.87) and early treatment with ticagrelor (RR 0.78; 95% CI 0.66–0.91) were associated with significant reductions in all-cause mortality risk compared with early treatment with clopidogrel (Supplementary Table S4). Heterogeneity of effects was low (I^2^ = 0%) and the overall inconsistency was not significant (*p* = 0.49). Early treatment with prasugrel ranked as the best intervention among all treatments for reducing all-cause mortality risk (*p*-score = 0.83) and delayed treatment with ticagrelor ranked as the worst intervention (*p*-score = 0.13) (Figure 2 and Supplementary Table S4).

#### Cardiovascular Mortality

Early treatment with clopidogrel was associated with a higher cardiovascular mortality risk compared with early treatment with prasugrel (RR 2.63; 95% CI 1.12–6.14) and early treatment with ticagrelor (RR 1.29; 95% CI 1.08–1.54) (Supplementary Table S5). Heterogeneity of effects was low (I^2^ = 0%) and the overall inconsistency was not significant (*p* = 0.54). Early treatment with prasugrel ranked as the best intervention for reducing cardiovascular mortality among all treatments (*p*-score = 0.92) and early treatment with clopidogrel ranked as the worst intervention (*p*-score = 0.12) (Figure 2 and Supplementary Table S4).

#### Myocardial Infarction

None of the comparisons showed significant effects on myocardial infarction (Supplementary Table S6). Heterogeneity of effects was moderate (I^2^ = 41%) and the overall inconsistency was not significant (*p* = 0.38). Delayed treatment with prasugrel ranked as the best intervention among all treatments for reducing myocardial infarction risk (*p*-score = 0.83) and early treatment with clopidogrel ranked as the worst intervention (*p*-score = 0.22) (Figure 2 and Supplementary Table S4).

#### Stent Thrombosis

Delayed treatment with clopidogrel was associated with a significantly higher stent thrombosis risk compared with early treatment with prasugrel (Supplementary Table S6). Heterogeneity of effects was low (I^2^ = 0%) and the overall inconsistency was not significant (*p* = 0.62). Early treatment with prasugrel ranked as the best intervention among other treatments for reducing stent thrombosis (*p*-score = 0.85) and early treatment with clopidogrel ranked as the worst intervention (*p*-score = 0.05) (Figure 2 and Supplementary Table S4).

#### Urgent Coronary Revascularization

None of the comparisons between treatment arms showed significant effects on urgent coronary revascularization risk (Supplementary Table S7). Heterogeneity and overall consistency could not be assessed. Early treatment with ticagrelor ranked as the best intervention for reducing urgent coronary revascularization risk (*p*-score = 0.93) and early treatment with prasugrel ranked as the worst intervention (*p*-score = 0.11) (Figure 2 and Supplementary Table S4).

#### Stroke

None of the comparisons between treatment arms showed a significant effect on stroke (Supplementary Table S6). Heterogeneity of effects was low (I^2^ = 0%) and the overall inconsistency was not significant (*p* = 0.99). Early treatment with prasugrel ranked as the best intervention for reducing stroke risk (*p*-score = 0.83) and delayed treatment with ticagrelor ranked as the worst intervention (*p*-score = 0.05) (Supplementary Table S4 and Figure 2).

### Sensitivity Analyses

After excluding the one trial evaluating only medically managed patients, (14) the ranking of best treatment strategies did not change for all primary and secondary outcomes (Supplementary Table S8). Considering only the delayed treatment with ticagrelor and prasugrel strategies of the DUBIUS trial (10), the ranking of best treatment strategies was also similar for all primary and secondary outcomes (Supplementary Table S9).

## Discussion

This network meta-analysis, including nine RCTs and ~40,000 patients with NSTE-ACS, comparing different treatment strategies of individual P2Y_12_ inhibitors and initiation times shows that: (1) the delayed initiation of treatment with prasugrel seems to be the most effective DAPT timing strategy for reducing MACE, (2) the early initiation of prasugrel is ranked as the best option for preventing most secondary cardiovascular outcomes but is associated with the highest increase in bleeding risk, and (3) a delayed initiation of treatment with clopidogrel is the safest option in terms of bleeding risk.

While there is clear evidence supporting the greater efficacy of the newer antiplatelet drugs, prasugrel and ticagrelor over clopidogrel in the treatment of ACS, at the expense of an increased bleeding risk (16, 21–24), the relative benefits and risks between these two drugs are less clear due to the differences in trial designs and, therefore, debated (4, 25). The optimal time for the initiation of P2Y_12_ inhibitor treatment in patients with NSTE-ACS is also a controversial issue (2, 4–6). The latest ESC NSTE-ACS guidelines recommend against the systematic early initiation of P2Y_12_ inhibitors. This recommendation is mainly based on the results of two RCTs, (6, 9) interpreted differently (4, 24–26). The ACCOAST trial (6), which compared early vs. delayed (“on the table”) administration of prasugrel in patients with NSTE-ACS in whom PCI was planned, showed no clinical benefit with the early initiation of prasugrel but an increased risk of bleeding although the difference in time between the early and delayed administration was only 4.3 h (6), shorter that can be expected in most centers in regular clinical practice. The ISAR-REACT 5 trial is an open label study comparing the efficacy and safety of ticagrelor vs. prasugrel in patients with ACS. Although the patients with NSTE-ACS allocated to the ticagrelor arm received the drug early and those allocated to the prasugrel arm received the drug “on the table,” the trial was not specifically designed to compare early vs. delayed P2Y_12_ initiation (9).

Given the shortage of direct evidence comparing the merits of the different time strategies for the initiation of the different P2Y_12_ inhibitors, there is a need to use indirect evidence to improve the available information. From this perspective, we have systematically reviewed all the direct and indirect evidence coming from RCTs to build this network meta-analysis and help clarifying the potential risks and benefits of the early vs. delayed administration of P2Y_12_ inhibitors in patients with NSTE-ACS, providing with a more precise effect estimation.

Our results confirm that there is no optimal timing for the initiation of P2Y_12_ inhibition. As expected, both early treatment with ticagrelor and early or delayed treatment with prasugrel are associated with reductions in MACE, cardiovascular mortality, and all-cause mortality risk compared with clopidogrel. However, in our analysis, delayed prasugrel initiation was the option ranked best for the reduction of MACE, the main endpoint of this review. This finding is partially driven by the results of the ISAR-REACT 5 trial (20) in which prasugrel showed a greater reduction in ischemic events compared with ticagrelor (20). Although this study has been criticized for being an open label study (4) and has been excluded from some meta-analyses (23), we have included it in our study to maximize the information comparing different timings of P2Y_12_ inhibitor initiation. The DUBIUS trial compared a delayed administration of oral P2Y_12_ inhibitors (prasugrel or ticagrelor, 1:1 randomization) vs. an early ticagrelor strategy in patients with NSTE-ACS. Early prasugrel initiation was not considered as a randomization arm in this trial. The study showed no significant differences in the MACE endpoint comparing delayed ticagrelor vs. delayed prasugrel, and the risk of bleeding was also comparable between both delayed treatments (10).

We have incorporated all trials including different times of P2Y_12_ initiation by any design. Compared with clopidogrel, prasugrel reduces ischemic events, such as MACE, stent thrombosis (27, 28), cardiovascular mortality, and stroke (29). However, the timing of administration makes an important difference. In our meta-analysis, while the early administration of prasugrel has a greater benefit in preventing ischemic events, it is associated with a significant increase in bleeding risk, not seen with a delayed administration, mostly “on the table.” For secondary outcomes, early treatment with prasugrel ranked as the best option for reducing all-cause mortality, cardiovascular mortality, stent thrombosis, and stroke risk while early ticagrelor ranked best only for reducing urgent coronary revascularization. Delayed ticagrelor initiation was associated with a lower bleeding risk compared with early ticagrelor treatment. The safest strategy associated with the lowest bleeding risk is delayed clopidogrel treatment, a finding consistent with the previous trials and meta-analyses (19, 23, 30), but with the poorest results in efficacy.

The network meta-analysis ranks delayed prasugrel as the best therapy for MACE with an acceptable risk of bleeding. Accordingly, it seems that the delayed initiation of prasugrel is the strategy associated with a more favorable benefit or risk balance when coronary angiography is anticipated to occur within a short time after the presentation, as recommended in the new ESC NSTE-ACS guidelines (2). Delayed ticagrelor initiation ranked better than early initiation for reducing MACE, with a lower risk of bleeding. However, it should be noted that only one trial (DUBIUS trial) specifically analyzed the efficacy of delayed administration of ticagrelor in the setting of NST-ACS (10), and the times of P2Y_12_ inhibitors administration (early vs. delayed) were also allocated randomly.

Our study may have clinical implications, as we have developed a ranking for all the potential combination of P2Y_12_ inhibitors and times of initiation in patients with NSTE-ACS for the main cardiovascular and safety outcomes. As stated in the current recommendations, (2) an effort should be made to better define the ischemic and hemorrhagic risk profile of patients with NSTE-ACS (2, 31–33), as well as to establish preference scenarios according to the therapeutic objectives. We cannot exclude the early initiation of P2Y_12_ inhibitors to be considered in patients in whom coronary angiography is going to be deferred by days for clinical or logistical reasons (not related to bleeding). In this case, early ticagrelor may be a reasonable option. The choice of P2Y_12_ inhibitor for delayed initiation may not be automatic. While prasugrel may be the first choice for delayed initiation, a number of patients may need treatment with clopidogrel due to contraindications or high bleeding risk. The delayed initiation of ticagrelor needs more evidence as only one study with no observed superiority has been published, and the available information suggests a superiority of delayed prasugrel initiation for the majority of endpoints.

This meta-analysis of randomized trials comparing different initiations of P2Y_12_ inhibitors increases our understanding of the strengths and weaknesses of the available evidence regarding the best timing to start DAP and pinpoints the need for more studies to properly define the optimal time of initiation of each P2Y_12_ inhibitor in patients with NSTE-ACS. Compared with previous meta-analyses (13, 24, 27, 29) our study presents a number of differences and advantages: i) it is the first meta-analysis focused on the timing of administration rather than on the comparison between drugs, ii) it is restricted to the initiation of P2Y_12_ inhibitors in patients with NSTE-ACS, probably the most controversial scenario for clinical decision-making (4, 5, 24, 34), iii) it summarizes all available information, including direct evidence and indirect estimations, providing a quantitative proxy of the potential benefits and risks of each therapeutic strategy with each P2Y_12_ inhibitor compared with the other options; iv) in contrast to the meta-analysis by Navarese et al. (23), it includes open label RCTs, as it is very difficult to have (and unlikely to happen) double blind face-to-face comparisons between all the P2Y_12_ inhibitors and time strategies; v) it is the only review that includes information on the delayed treatment with ticagrelor; vi) it includes patients of all ages, not only the elderly (24), and vii) it includes an analysis of cardiovascular mortality, a relevant endpoint not available in all the studies (24, 27). However, this review should be interpreted with caution considering a number of limitations. First, our study did not use patient-level analysis but was performed at the trial level. Many studies included both patients with ST elevation myocardial infarction (STEMI) and non-STEMI and randomization did not take into account the type of ACS. Second, there are differences among trials in the definition of some endpoints, particularly bleeding, and therefore, the estimates are for slightly different event risks. Third, the differences in the duration of P2Y_12_ inhibitor treatment between the two arms may have had an impact on bleeding and anti-ischemic efficacy but this was not available in some studies. Fourth, although we found no evidence of statistical inconsistency, a moderate to high heterogeneity of effects was found in our primary outcomes, which may be expected due to the different study designs, population types, revascularization strategies, and follow-up durations. Fifth, we could not include the analysis of net adverse clinical events as this composite endpoint was only reported in three RCTs. Sixth, it was not possible to have subgroup analyses due to the paucity of data. Finally, since data on delayed treatment with ticagrelor were only reported in one RCT, our findings for this option should be interpreted with caution.

## Conclusions

Considering all the direct and indirect evidence from RCTs, the delayed administration of prasugrel for DAPT initiation seems to be the most effective strategy to reduce MACE in patients with NSTE-ACS. Early prasugrel seems the best option to reduce most secondary cardiovascular outcomes but is associated with the highest bleeding risk. The delayed initiation of clopidogrel is the safest strategy but with poor results in preventing all cardiovascular outcomes. Adequately sized RCTs addressing specifically these questions are needed to define clearly which P2Y_12_ inhibitor should be started and when in the different clinical scenarios in patients with NSTE-ACS.

## Data Availability Statement

The original contributions presented in the study are included in the article/Supplementary Material, further inquiries can be directed to the corresponding author/s.

## Author Contributions

All authors listed have made a substantial, direct, and intellectual contribution to the work and approved it for publication.

## Funding

Unrelated to the study, LV receives research funding from the Instituto de Salud Carlos III, Spain (CM20/00104). HB receives research funding from the Instituto de Salud Carlos III, Spain (PIE16/00021 & PI17/01799), Sociedad Española de Cardiología, Astra-Zeneca, Bayer, PhaseBio and Novartis; has received consulting fees from Astra-Zeneca, Novartis; speaking fees from Novartis and is a scientific advisor for MEDSCAPE-the heart.og.

## Conflict of Interest

GT reviewed payment or honoraria for lectures, presentations, speakers' bureaus, manuscript writing, or educational events from Daichii Sankyo and Astra Zeneca. MM reviewed payment or honoraria for lectures, presentations, speakers' bureaus, manuscript writing, or educational events from Daichii Sankyo and Astra Zeneca. The remaining authors declare that the research was conducted in the absence of any commercial or financial relationships that could be construed as a potential conflict of interest.

## Publisher's Note

All claims expressed in this article are solely those of the authors and do not necessarily represent those of their affiliated organizations, or those of the publisher, the editors and the reviewers. Any product that may be evaluated in this article, or claim that may be made by its manufacturer, is not guaranteed or endorsed by the publisher.

## Abbreviations

ACS: Acute coronary syndrome
DAPT: Dual antiplatelet therapy
HR: Hazard ratio
MACE: Major adverse cardiovascular events
NST-ACS: Non-ST-segment elevation acute coronary syndrome
PCI: Percutaneous coronary intervention
RCT: Randomized controlled trials
RR: Risk ratio.

## References

[1] Valgimigli M Bueno H Byrne RA Collet J-P Costa F Jeppsson A . 2017 ESC focused update on dual antiplatelet therapy in coronary artery disease developed in collaboration with EACTS: the task force for dual antiplatelet therapy in coronary artery disease of the European Society of Cardiology (ESC) and of the European Association for Cardio-Thoracic Surgery (EACTS). Eur Heart J. (2018) 39:213–60. 10.1093/eurheartj/ehx638 28886622

[2] Collet J-P Thiele H Barbato E Barthélémy O Bauersachs J Bhatt DL . 2020 ESC Guidelines for the management of acute coronary syndromes in patients presenting without persistent ST-segment elevation: The Task Force for the management of acute coronary syndromes in patients presenting without persistent ST-segment elevation of the European Society of Cardiology (ESC). Eur Heart J. (2020) 42:1289–367. 10.1093/eurheartj/ehaa895 32860058

[3] Amsterdam Ezra A Wenger Nanette K Brindis Ralph G Casey Donald E Ganiats Theodore G Holmes David R . 2014 AHA/ACC guideline for the management of patients with non–ST-elevation acute coronary syndromes. J Am Coll Cardiol. (2014) 64:e139–228. 10.1016/j.jacc.2014.09.017 25260718

[4] Collet J-P Thiele H Giannitsis E Sibbing D Barthélémy O Bauersachs J . Debate: Prasugrel rather than ticagrelor is the preferred treatment for NSTE-ACS patients who proceed to PCI and pretreatment should not be performed in patients planned for an early invasive strategy. Eur Heart J England. (2021) 42:2973–2985. 10.1093/eurheartj/ehab277 34110420

[5] Sibbing D Kastrati A Berger PB. Pre-treatment with P2Y12 inhibitors in ACS patients: who, when, why, and which agent? Eur Heart J. (2016) 37:1284–95. 10.1093/eurheartj/ehv717 26712838

[6] Montalescot G Bolognese L Dudek D Goldstein P Hamm C Tanguay J-F . Pretreatment with prasugrel in non–ST-segment elevation acute coronary syndromes. N Engl J Med. (2013) 369:999–1010. 10.1056/NEJMoa1308075 23991622

[7] Hamm CW Bassand J-P Agewall S Bax J Boersma E Bueno H . ESC Guidelines for the management of acute coronary syndromes in patients presenting without persistent ST-segment elevation: The Task Force for the management of acute coronary syndromes (ACS) in patients presenting without persistent ST-segment elevation of the European Society of Cardiology (ESC). Eur Heart J. (2011) 32:2999–3054. 10.1093/eurheartj/ehr236 21873419

[8] Roffi M Patrono C Collet J-P Mueller C Valgimigli M Andreotti F . 2015 ESC Guidelines for the management of acute coronary syndromes in patients presenting without persistent ST-segment elevation: Task Force for the Management of Acute Coronary Syndromes in Patients Presenting without Persistent ST-Segment Elevation of the European Society of Cardiology (ESC). Eur Heart J. (2016) 37:267–315. 10.1093/eurheartj/ehv320 26320110

[9] Schüpke S Neumann F-J Menichelli M Mayer K Bernlochner I Wöhrle J . Ticagrelor or prasugrel in patients with acute coronary syndromes. N Engl J Med. (2019) 381:1524–34. 10.1056/NEJMoa1908973 31475799

[10] Tarantini G Mojoli M Varbella F Caporale R Rigattieri S Andò G . Timing of Oral P2Y12 inhibitor administration in patients with non-ST-segment elevation acute coronary syndrome. J Am Coll Cardiol. (2020) 76:2450–9. 10.1016/j.jacc.2020.08.053 32882390

[11] Hutton B Salanti G Caldwell DM Chaimani A Schmid CH Cameron C . The PRISMA extension statement for reporting of systematic reviews incorporating network meta-analyses of health care interventions: checklist and explanations. Ann Intern Med. (2015) 162:777–84. 10.7326/M14-2385 26030634

[12] Higgins JPT Jackson D Barrett JK Lu G Ades AE White IR. Consistency and inconsistency in network meta-analysis: concepts and models for multi-arm studies. Res Synth Methods. (2012) 3:98–110. 10.1002/jrsm.1044 26062084 PMC4433772

[13] Rücker G Schwarzer G. Ranking treatments in frequentist network meta-analysis works without resampling methods. BMC Med Res Methodol. (2015) 15:58. 10.1186/s12874-015-0060-8 26227148 PMC4521472

[14] Roe MT Armstrong PW Fox KAA White HD Prabhakaran D Goodman SG . Prasugrel versus clopidogrel for acute coronary syndromes without revascularization. N Engl J Med. (2012) 367:1297–309. 10.1056/NEJMoa1205512 22920930

[15] De Servi S Goedicke J Schirmer A Widimsky P. Clinical outcomes for prasugrel versus clopidogrel in patients with unstable angina or non-ST-elevation myocardial infarction: an analysis from the TRITON-TIMI 38 trial. Eur Heart J Acute Cardiovasc Care England. (2014) 3:363–72. 10.1177/2048872614534078 24818952

[16] Lindholm D Varenhorst C Cannon CP Harrington RA Himmelmann A Maya J . Ticagrelor vs. clopidogrel in patients with non-ST-elevation acute coronary syndrome with or without revascularization: results from the PLATO trial. Eur Heart J. (2014) 35:2083–93. 10.1093/eurheartj/ehu160 24727884 PMC4132637

[17] Bonello L Laine M Cluzel M Frere C Mancini J Hasan A . Comparison of ticagrelor versus prasugrel to prevent periprocedural myonecrosis in acute coronary syndromes. Am J Cardiol. (2015) 116:339–43. 10.1016/j.amjcard.2015.04.050 26037292

[18] Savonitto S Ferri LA Piatti L Grosseto D Piovaccari G Morici N . Comparison of reduced-dose prasugrel and standard-dose clopidogrel in elderly patients with acute coronary syndromes undergoing early percutaneous revascularization. Circulation. (2018) 137:2435–45. 29459361 10.1161/CIRCULATIONAHA.117.032180

[19] Gimbel M Qaderdan K Willemsen L Hermanides R Bergmeijer T Vrey E de . Clopidogrel versus ticagrelor or prasugrel in patients aged 70 years or older with non-ST-elevation acute coronary syndrome (POPular AGE): the randomised, open-label, non-inferiority trial. Lancet. (2020) 395:1374–81. 10.1016/S0140-6736(20)30325-1 32334703

[20] Valina C Neumann F-J Menichelli M Mayer K Wöhrle J Bernlochner I . Ticagrelor or prasugrel in patients with non-ST-segment elevation acute coronary syndromes. J Am Coll Cardiol United States. (2020) 76:2436–46. 10.1016/j.jacc.2020.09.584 33213722

[21] Wiviott SD Braunwald E McCabe CH Montalescot G Ruzyllo W Gottlieb S. Prasugrel versus clopidogrel in patients with acute coronary syndromes. N Engl J Med. (2007) 357:2001–15. 10.1056/NEJMoa0706482 17982182

[22] Wallentin L Becker RC Budaj A Cannon CP Emanuelsson H Held C . Ticagrelor versus clopidogrel in patients with acute coronary syndromes. N Engl J Med. (2009) 361:1045–57. 10.1056/NEJMoa0904327 19717846

[23] Navarese EP Khan SU Kołodziejczak M Kubica J Buccheri S Cannon CP . Comparative efficacy and safety of oral P2Y12 inhibitors in acute coronary syndrome. Circulation. (2020) 142:150–60. 10.1161/CIRCULATIONAHA.120.046786 32468837 PMC7489363

[24] Montalto C Morici N Munafò AR Mangieri A Mandurino-Mirizzi A D'Ascenzo F . Optimal P2Y12 inhibition in older adults with acute coronary syndromes: a network meta-analysis of randomized controlled trials. Eur Heart J Cardiovasc Pharm. (2020) 8:20–7. 10.1093/ehjcvp/pvaa101 32835355

[25] Kow CS Zaihan AF Hasan SS. Prasugrel over ticagrelor in non-ST-elevation acute coronary syndromes: is it justified? Eur Heart J. (2021) 42:2609–10. 10.1093/eurheartj/ehaa880 33205147

[26] Kirtane AJ. ISAR-REACT 5 revisited through the lens of a postrandomization subgroup. JAMA Cardiol. (2021) 6:1129. 10.1001/jamacardio.2021.2238 34190958

[27] Chatterjee S Ghose A Sharma A Guha G Mukherjee D Frankel R. Comparing newer oral anti-platelets prasugrel and ticagrelor in reduction of ischemic events-evidence from a network meta-analysis. J Thromb Thrombol. (2012) 36. 10.1007/s11239-012-0838-z 23212803

[28] Shah R Rashid A Hwang I Fan T-HM Khouzam RN Reed GL. Meta-analysis of the relative efficacy and safety of oral P2Y12 inhibitors in patients with acute coronary syndrome. Am J Cardiol. (2017) 119:1723–8. 10.1016/j.amjcard.2017.03.011 28385176

[29] Fei Y Lam CK Cheung BMY. Efficacy and safety of newer P2Y12 inhibitors for acute coronary syndrome: a network meta-analysis. Sci Rep. (2020) 10:16794. 10.1038/s41598-020-73871-x 33033323 PMC7545197

[30] Huynh K. Clopidogrel is a favourable alternative to ticagrelor in older patients with NSTE-ACS. Nature Rev Cardiol. (2020) 17:384–384. 10.1038/s41569-020-0393-9 32404962

[31] Urban P Mehran R Colleran R Angiolillo DJ Byrne RA Capodanno D . Defining high bleeding risk in patients undergoing percutaneous coronary intervention: a consensus document from the Academic Research Consortium for High Bleeding Risk. Eur Heart J. (2019) 40:2632–53. 10.1093/eurheartj/ehz372 31116395 PMC6736433

[32] Antoniou S Colicchia M Guttmann OP Rathod KS Wright P Fhadil S . Risk scoring to guide antiplatelet therapy post-percutaneous coronary intervention for acute coronary syndrome results in improved clinical outcomes. Eur Heart J Qual Care Clin Outcomes. (2018) 4:283–9. 10.1093/ehjqcco/qcx041 29126112

[33] Bing R Goodman SG Yan AT Fox K Gale CP Hyun K . Use of clinical risk stratification in non-ST elevation acute coronary syndromes: an analysis from the CONCORDANCE registry. Eur Heart J Qual Care Clin Outcomes. (2018) 4:309–17. 10.1093/ehjqcco/qcy002 29438470

[34] Marco V. Pretreatment with P2Y12 inhibitors in Non–ST-Segment–Elevation Acute Coronary Syndrome is clinically justified. Circulation. (2014) 130:1891–1903. 10.1161/CIRCULATIONAHA.114.011319 25403595

