# Supplementary material for: Early vs. Delayed Initiation of Treatment With P2Y12 Inhibitors in Patients With Non-ST-Segment Elevation Acute Coronary Syndrome: A Systematic Review and Network Meta-Analysis of Randomized Controlled Trials
Source: Front Cardiovasc Med. 2022 Apr 28;9:862452. doi: 10.3389/fcvm.2022.862452 (PMC9095971; doi:10.3389/fcvm.2022.862452)
Supplement: Supplementary file 1 [file Data_Sheet_1.docx]

**SUPPLEMENTARY DATA**

**Table S1. Search strategy (at January 09, 2021).**

| **PubMed (1152 hits)**  (clopidogrel[tiab] OR prasugrel[tiab] OR ticagrelor[tiab] OR P2Y12[tiab]) AND (“unstable angina”[tiab] OR “non-ST-segment elevation”[tiab] OR “non-ST elevation”[tiab] OR “acute coronary syndrome”[tiab] OR “acute coronary syndromes”[tiab]) AND (random*[tiab] OR placebo[tiab] OR “clinical trial”[tiab]) |
| --- |
| **EMBASE (1743 hits)**  ('clopidogrel':ti,ab OR 'prasugrel':ti,ab OR 'ticagrelor':ti,ab OR 'p2y12':ti,ab) AND ('unstable angina':ti,ab OR 'non-st-segment elevation':ti,ab OR 'non-st elevation':ti,ab OR 'acute coronary syndrome':ti,ab OR 'acute coronary syndromes':ti,ab) AND ('random*':ti,ab OR 'placebo':ti,ab OR 'clinical trial':ti,ab) |
| **Scopus (1364 hits)**  TITLE-ABS ((“clopidogrel” OR “prasugrel” OR “ticagrelor” OR “P2Y12”) AND (“unstable angina” OR “non-ST-segment elevation” OR “non-ST elevation” OR “acute coronary syndrome” OR “acute coronary syndromes”) AND (“random*” OR “placebo” OR “clinical trial”)) |
| **Web of Science (957 hits)**  TI=((“clopidogrel” OR “prasugrel” OR “ticagrelor” OR “P2Y12”) AND (“unstable angina” OR “non-ST-segment elevation” OR “non-ST elevation” OR “acute coronary syndrome” OR “acute coronary syndromes”) AND (“random*” OR “placebo” OR “clinical trial”)) OR AB=((“clopidogrel” OR “prasugrel” OR “ticagrelor” OR “P2Y12”) AND (“unstable angina” OR “non-ST-segment elevation” OR “non-ST elevation” OR “acute coronary syndrome” OR “acute coronary syndromes”) AND (“random*” OR “placebo” OR “clinical trial”)) |
| **CENTRAL (1459 hits)**  (clopidogrel OR prasugrel OR ticagrelor OR P2Y12) AND (“unstable angina” OR “non-ST-segment elevation” OR “non-ST elevation” OR “acute coronary syndrome” OR “acute coronary syndromes”) in Title and Abstract |

**Table S2. Definitions of MACE and bleeding**

| **Study** | **Efficacy endpoint** | **Key safety endpoint** |
| --- | --- | --- |
|  | **MACE** | **Bleeding** |
| Bonello, 2015 | Cardiovascular death, myocardial infarction, urgent revascularization, or stroke | BARC >2 |
| De Servi, 2014 | Death from cardiovascular causes, nonfatal myocardial infarction, or nonfatal stroke | Non-CABG TIMI major bleeding |
| Montalescot, 2013 | Death from cardiovascular causes, myocardial infarction, stroke, urgent revascularization, or glycoprotein IIb/IIIa bailout | All CABG-related or non–CABG-related TIMI major bleeding |
| Gimbel, 2020 | Cardiovascular death, myocardial infarction, stroke, PLATO major or minor bleeding | PLATO major or minor bleeding |
| Lindholm, 2014 | Cardiovascular death, myocardial infarction (excluding silent infarctions), or stroke | PLATO major bleeding |
| Valina, 2020 | Death, myocardial infarction, or stroke | BARC 3, 4, or 5 |
| Tarantini, 2020 | Death from vascular causes (cardiovascular or cerebrovascular death, any death without another known cause), non-fatal myocardial infarction, non-fatal stroke, major or fatal bleeding (BARC 3, 4, or 5) | BARC 3, 4, or 5 |
| Savonitto, 2018 | All-cause mortality, myocardial infarction, disabling stroke, and rehospitalization for cardiovascular causes or bleeding within 1 year | - |
| Roe, 2012 | Cardiovascular death, myocardial infarction or stroke | TIMI major bleeding not related to CABG |

MACE, major adverse cardiovascular events; BARC, bleeding academic research consortium; CABG, coronary artery bypass grafting; TIMI, thrombolysis in myocardial infarction; PLATO, platelet inhibition and patient outcomes; GUSTO, global use of strategies to open occluded arteries.

**Table S3. Demographic and clinical characteristics of included patients.**

| **Study** | **Arms** | **Timing** | **Dosage** | **Age, years** | **Male** | **HTN** | **DM** | **Previous MI** | **Previous PCI** | **Previous CABG** | **Type of**  **NSTE-ACS** |
| --- | --- | --- | --- | --- | --- | --- | --- | --- | --- | --- | --- |
| **Bonello,**  **2015** | Ticagrelor | Early | 180 mg LD, then 90 mg BID | 61.5±10.4 | 70% | 53% | 29% | 11% | 12% | 3% | Unstable angina (46%), NSTEMI (54%) |
|  | Prasugrel | Delayed | 60 mg LD, then 10mg QD | 60 ± 9.6 | 80% | 58% | 41% | 12% | 12% | 3% | Unstable angina (54%), NSTEMI (46%) |
| **De Servi,**  **2014** | Prasugrel | Delayed | 60 mg LD, then 10mg QD | 61.5±11.2 | 74% | 69% | 25% | 21% | 16% | 10% | Unstable angina (25%), NSTEMI (75%) |
|  | Clopidogrel | Delayed | 300 mg LD, then 75 mg QD | 61.3±11.3 | 72% | 69% | 24% | 20% | 16% | 9% | Unstable angina (25%), NSTEMI (75%) |
| **Montalescot, 2013** | Prasugrel | Early | 30 mg LD, then 10 mg QD (5 mg in ≥75 years or body weight <60 kg) | 63.8 | 73% | 63% | 20% | 14% | 16% | 5% | NSTEMI (100%) |
|  | Prasugrel | Delayed | 60 mg LD, then 10 mg QD (5 mg in ≥75 years or body weight <60 kg) | 63.6 | 72% | 61% | 20% | 15% | 17% | 5% | NSTEMI (100%) |
| **Gimbel,**  **2020** | Ticagrelor | Early | 180 mg LD, then 90 mg BID | 77 (73-82) | 65% | 73% | 30% | 27% | 24% | 17% | Unstable angina (11%), NSTEMI (86%), other (4%) |
|  | Clopidogrel | Early | 300-600 mg LD, then 75 mg QD | 77 (73-81) | 63% | 73% | 29% | 24% | 20% | 17% | Unstable angina (11%), NSTEMI (100%), other (3%) |
| **Lindholm,**  **2014** | Ticagrelor | Early | 180 mg LD, then 90 mg BID | 64 (56-72) | 69% | 70% | 29% | 25% | 17% | 8% | Unstable angina (27%), NSTEMI (65%), STEMI (8%) |
|  | Clopidogrel | Early | 300-600 mg LD, then 75 mg QD | 64 (56-72) | 68% | 70% | 28% | 26% | 17% | 9% | Unstable angina (28%), NSTEMI (64%), STEMI (8%) |
| **Valina,**  **2020** | Ticagrelor | Early | 180 mg LD, then 90 mg BID | 66.0 ±11.7 | 74% | 78% | 25% | 18% | 28% | 7% | Unstable angina (21%), NSTEMI (79%) |
|  | Prasugrel | Delayed | 60 mg LD, then 10 mg QD (5 mg in elderly) | 65.6±1 2.0 | 74% | 75% | 24% | 19% | 29% | 9% | Unstable angina (22%), NSTEMI (78%) |
| **Tarantini,**  **2020** | Ticagrelor | Early | 180 mg LD, then 90 mg BID | 64 (57-72) | 75% | 68% | 24% | 19% | 20% | 5% | Unstable angina (21%), NSTEMI (79%) |
|  | Prasugrel | Delayed | Prasugrel 60mg LD, then 5 or 10mg QD | 65 ± 8 | 84% | 66% | 25% | 15% | 16% | 4% | Unstable angina (18%), NSTEMI (79%) |
|  | Ticagrelor | Delayed | Ticagrelor 180 mg LD, then 90mg BID | 65 ± 8 | 82% | 74% | 25% | 17% | 17% | 3% | Unstable angina (19%), NSTEMI (77%) |
| **Savonitto,**  **2018** | Prasugrel | Delayed | 60 mg LD, then 5 mg QD | 80 (77-83) | 63% | 82% | 32% | 26% | 24% | 12% | Unstable angina (17%), NSTEMI (83%) |
|  | Clopidogrel | Delayed | 300-600 mg LD, then 75 mg QD |  |  |  |  |  |  |  | Unstable angina (19%), NSTEMI (81%) |
| **Roe,**  **2012** | Prasugrel | Delayed | 30 mg LD, then 5 or 10 mg QD | 66 (58 - 74) | 61% | 82% | 38% | 43% | 26% | 15% | Unstable angina (29.6%), NSTEMI (70.4%) |
|  | Clopidogrel | Delayed | 300 mg, then 75 mg QD | 66 (59 - 73) | 61% | 82% | 38% | 43% | 27% | 16% | Unstable angina (30.6%), NSTEMI (69.4%) |

DM, diabetes mellitus; HTN, hypertension; MI, myocardial infarction; PCI, percutaneous coronary intervention; CABG, coronary artery bypass grafting; NSTE-ACS, non-ST-segment elevation acute coronary syndrome; LD, load dose; QD, once a day; BID, twice a day; NSTEMI, non-ST-segment elevation myocardial infarction.

**Table S4. Raking of P2Y12 inhibitors per outcome. A higher p-score means such a treatment ranks better than others with lower p-scores for a given outcome.**

|  | **Outcomes** | | | | | | | | | | |
| --- | --- | --- | --- | --- | --- | --- | --- | --- | --- | --- | --- |
| ***Treatment arms*** | **MACE (HR)** | **MACE** | **Bleeding (HR)** | **Bleeding** | **All-cause mortality** | **CV mortality** | **Myocardial infarction** | **Stent thrombosis** | **Urgent coronary revascularization** | **Stroke** |  |
| **Early clopidogrel** | 0.04 | 0.06 | 0.32 | 0.61 | 0.17 | 0.12 | 0.22 | 0.05 | 0.80 | 0.59 |  |
| **Delayed clopidogrel** | 0.51 | 0.42 | 0.84 | 0.88 | 0.64 | 0.52 | 0.49 | 0.40 | - | 0.45 |  |
| **Early prasugrel** | 0.79 | 0.67 | 0.06 | 0.02 | 0.83 | 0.92 | 0.67 | 0.85 | 0.11 | 0.83 |  |
| **Delayed prasugrel** | 0.80 | 0.70 | 0.55 | 0.50 | 0.79 | 0.67 | 0.83 | 0.59 | 0.31 | 0.51 |  |
| **Early ticagrelor** | 0.29 | 0.28 | 0.47 | 0.35 | 0.43 | 0.41 | 0.43 | 0.41 | 0.93 | 0.56 |  |
| **Delayed ticagrelor** | 0.57 | 0.87 | 0.76 | 0.63 | 0.13 | 0.36 | 0.36 | 0.71 | 0.34 | 0.05 |  |

MACE, major adverse cardiovascular events; HR, hazard ratio; CV, cardiovascular.

Cells in green and red are the highest and lowest p-score values, respectively.

**Table S5. League table of the effects of P2Y12 inhibitors expressed as risk ratio with their 95% CIs on all-cause mortality (white cells) and cardiovascular mortality (gray cells).**

| Delayed clopidogrel | 0.65 (0.38-1.11) | 1.06 (0.93-1.21) | 1.70 (0.86-3.36) | 0.65 (0.07-6.39) | 0.83 (0.50-1.39) |
| --- | --- | --- | --- | --- | --- |
| **1.69 (1.06-2.70)** | Early clopidogrel | 1.64 (0.97-2.77) | **2.63 (1.12-6.14)** | 1.00 (0.10-9.72) | **1.29 (1.08-1.54)** |
| 0.94 (0.82-1.07) | **0.56 (0.35-0.87)** | Delayed prasugrel | 1.60 (0.82-3.13) | 0.61 (0.06-6.00) | 0.78 (0.48-1.29) |
| 0.74 (0.29-1.88) | 0.43 (0.15-1.22) | 0.78 (0.31-1.98) | Early prasugrel | 0.38 (0.04-4.12) | 0.49 (0.21-1.12) |
| 3.27 (0.54-20.00) | 1.93 (0.32-11.65) | 3.48 (0.57-21.14) | 4.44 (0.58-33.75) | Delayed ticagrelor | 1.28 (0.13-12.33) |
| 1.32 (0.85-2.04) | **0.78 (0.66-0.91)** | 1.40 (0.92-2.13) | 1.79 (0.65-4.94) | 0.40 (0.07-2.41) | Early ticagrelor |

CIs, confidence intervals; MACE, major adverse cardiovascular events. For hazard ratios of all-cause mortality and cardiovascular mortality the comparison is row versus column (comparator). Effects in bold are statistically significant.

**Table S6. League table of the effects of P2Y12 inhibitors expressed as risk ratio with their 95% CIs on myocardial infarction (white cells) and stent thrombosis (gray cells).**

| Delayed clopidogrel | 0.10 (0.01-2.87) | 1.60 (0.38-6.64) | 4.08 (0.46-35.75) | 2.96 (0.11-76.96) | 1.06 (0.18-6.32) |
| --- | --- | --- | --- | --- | --- |
| 1.23 (0.75-2.04) | Early clopidogrel | 16.67 (0.76-365.06) | **42.53 (1.29-1400.68)** | 30.89 (0.52-1820.96) | 11.04 (0.61-199.20) |
| 0.86 (0.72-1.02) | 0.69 (0.43-1.11) | Delayed prasugrel | 2.55 (0.50-13.14) | 1.85 (0.10-34.70) | 0.66 (0.23-1.95) |
| 0.91 (0.63-1.31) | 0.74 (0.42-1.30) | 1.06 (0.77-1.46) | Early prasugrel | 0.73 (0.03-20.85) | 0.26 (0.04-1.85) |
| 1.29 (0.34-4.92) | 1.04 (0.28-3.91) | 1.51 (0.40-5.68) | 1.42 (0.36-5.54) | Delayed ticagrelor | 0.36 (0.02-6.32) |
| 1.10 (0.70-1.73) | 0.89 (0.72-1.11) | 1.29 (0.85-1.95) | 1.21 (0.72-2.04) | 0.85 (0.23-3.13) | Early ticagrelor |

CIs, confidence intervals; MACE, major adverse cardiovascular events. For hazard ratios of MACE and bleeding the comparison is row versus column (comparator). Effects in bold are statistically significant.

**Table S7. League table of the effects of P2Y12 inhibitors expressed as risk ratio with their 95% CIs on stroke (white cells) and urgent coronary revascularization (gray cells).**

| Delayed clopidogrel | - | - | - | - | - |
| --- | --- | --- | --- | --- | --- |
| 0.88 (0.36-2.12) | Early clopidogrel | 0.12 (0.02-0.70) | 0.09 (0.01-0.58) | 0.14 (0.02-0.83) | 1.34 (0.57-3.15) |
| 0.96 (0.74-1.25) | 1.09 (0.47-2.54) | Delayed prasugrel | 0.74 (0.39-1.41) | 1.16 (0.39-3.38) | 11.31 (2.37-54.05) |
| 0.61 (0.25-1.45) | 0.69 (0.21-2.25) | 0.63 (0.27-1.45) | Early prasugrel | 1.56 (0.45-5.44) | 15.24 (2.81-82.63) |
| 5.51 (0.59-51.06) | 6.26 (0.70-55.73) | 5.72 (0.63-52.17) | 9.08 (0.85-96.47) | Delayed ticagrelor | 9.78 (1.99-48.13) |
| 0.90 (0.39-2.05) | 1.02 (0.74-1.40) | 0.93 (0.43-2.03) | 1.48 (0.47-4.64) | 0.16 (0.02-1.42) | Early ticagrelor |

CIs, confidence intervals; MACE, major adverse cardiovascular events. For hazard ratios of MACE and bleeding the comparison is row versus column (comparator). Effects in bold are statistically significant.

**Table S8. Sensitivity analysis excluding Roe et al. trial.**

|  |  | |  | |  | | **Outcomes** | | | | | | |
| --- | --- | --- | --- | --- | --- | --- | --- | --- | --- | --- | --- | --- | --- |
| ***Treatment arms*** | **MACE (HR)** | **MACE** | | **Bleeding (HR)** | | **Bleeding** | | **All-cause mortality** | **CV mortality** | **Myocardial infarction** | **Stent thrombosis** | **Urgent coronary revascularization** | **Stroke** |
| **Early clopidogrel** | 0.04 | 0.06 | | 0.32 | | 0.61 | | 0.21 | 0.12 | 0.18 | 0.05 | 0.80 | 0.59 |
| **Delayed clopidogrel** | 0.51 | 0.35 | | 0.84 | | 0.88 | | - | 0.58 | 0.38 | 0.40 | - | 0.45 |
| **Early prasugrel** | 0.79 | 0.70 | | 0.06 | | 0.02 | | 0.86 | 0.91 | 0.73 | 0.85 | 0.11 | 0.83 |
| **Delayed prasugrel** | 0.80 | 0.71 | | 0.55 | | 0.50 | | 0.79 | 0.62 | 0.86 | 0.59 | 0.31 | 0.51 |
| **Early ticagrelor** | 0.29 | 0.30 | | 0.47 | | 0.35 | | 0.51 | 0.40 | 0.47 | 0.41 | 0.93 | 0.56 |
| **Delayed ticagrelor** | 0.57 | 0.87 | | 0.76 | | 0.63 | | 0.14 | 0.36 | 0.37 | 0.71 | 0.34 | 0.05 |

MACE, major adverse cardiovascular events; HR, hazard ratio; CV, cardiovascular.

Cells in green and red are the highest and lowest p-score values, respectively.

**Table S9. Sensitivity analysis considering only the delayed treatment with ticagrelor and prasugrel groups of the Tarantini et al. trial.**

|  | **Outcomes** | | | | | | | | | |
| --- | --- | --- | --- | --- | --- | --- | --- | --- | --- | --- |
| **Treatment arms** | **MACE (HR)** | **MACE** | **Bleeding (HR)** | **Bleeding** | **All-cause mortality** | **CV mortality** | **Myocardial infarction** | **Stent thrombosis** | **Urgent coronary revascularization** | **Stroke** |
| **Early clopidogrel** | 0.06 | 0.05 | 0.34 | 0.55 | 0.12 | 0.09 | 0.16 | 0.04 | - | 0.57 |
| **Delayed clopidogrel** | 0.50 | 0.43 | 0.81 | 0.85 | 0.61 | 0.50 | 0.47 | 0.40 | - | 0.43 |
| **Early prasugrel** | 0.76 | 0.66 | 0.07 | 0.02 | 0.80 | 0.89 | 0.64 | 0.83 | 0.21 | 0.82 |
| **Delayed prasugrel** | 0.78 | 0.69 | 0.53 | 0.51 | 0.76 | 0.64 | 0.80 | 0.58 | 0.61 | 0.49 |
| **Early ticagrelor** | 0.29 | 0.25 | 0.49 | 0.30 | 0.37 | 0.36 | 0.34 | 0.37 | - | 0.54 |
| **Delayed ticagrelor** | 0.61 | 0.91 | 0.76 | 0.75 | 0.34 | 0.52 | 0.58 | 0.78 | 0.68 | 0.14 |

MACE, major adverse cardiovascular events; HR, hazard ratio; CV, cardiovascular.

Cells in green and red are the highest and lowest p-score values, respectively.

**Figure S1. Risk of Bias 2.0 tool for risk assessment of included randomized controlled trials.**

**Figure S2. Network geometries for primary and secondary outcomes.**

**
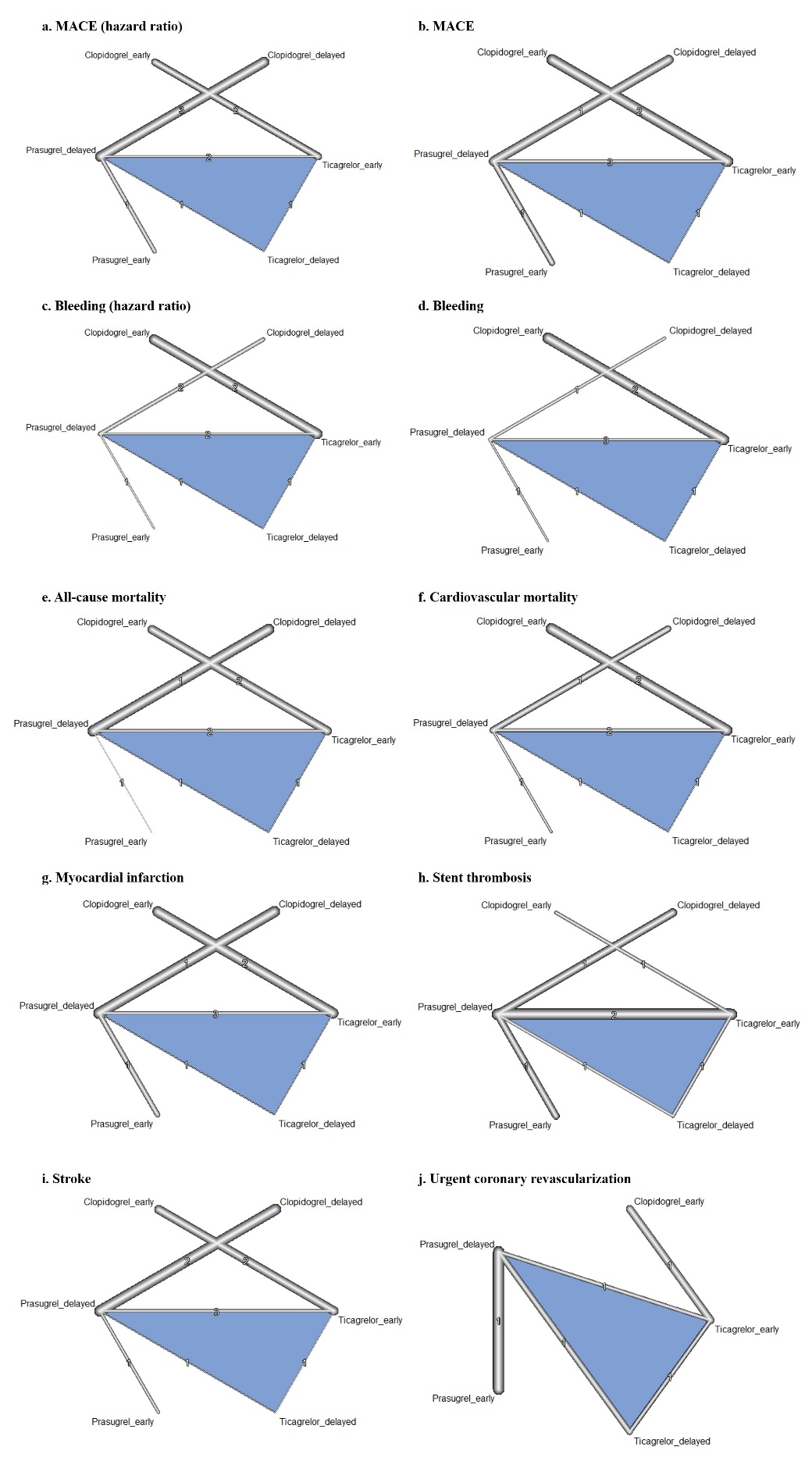
**

**
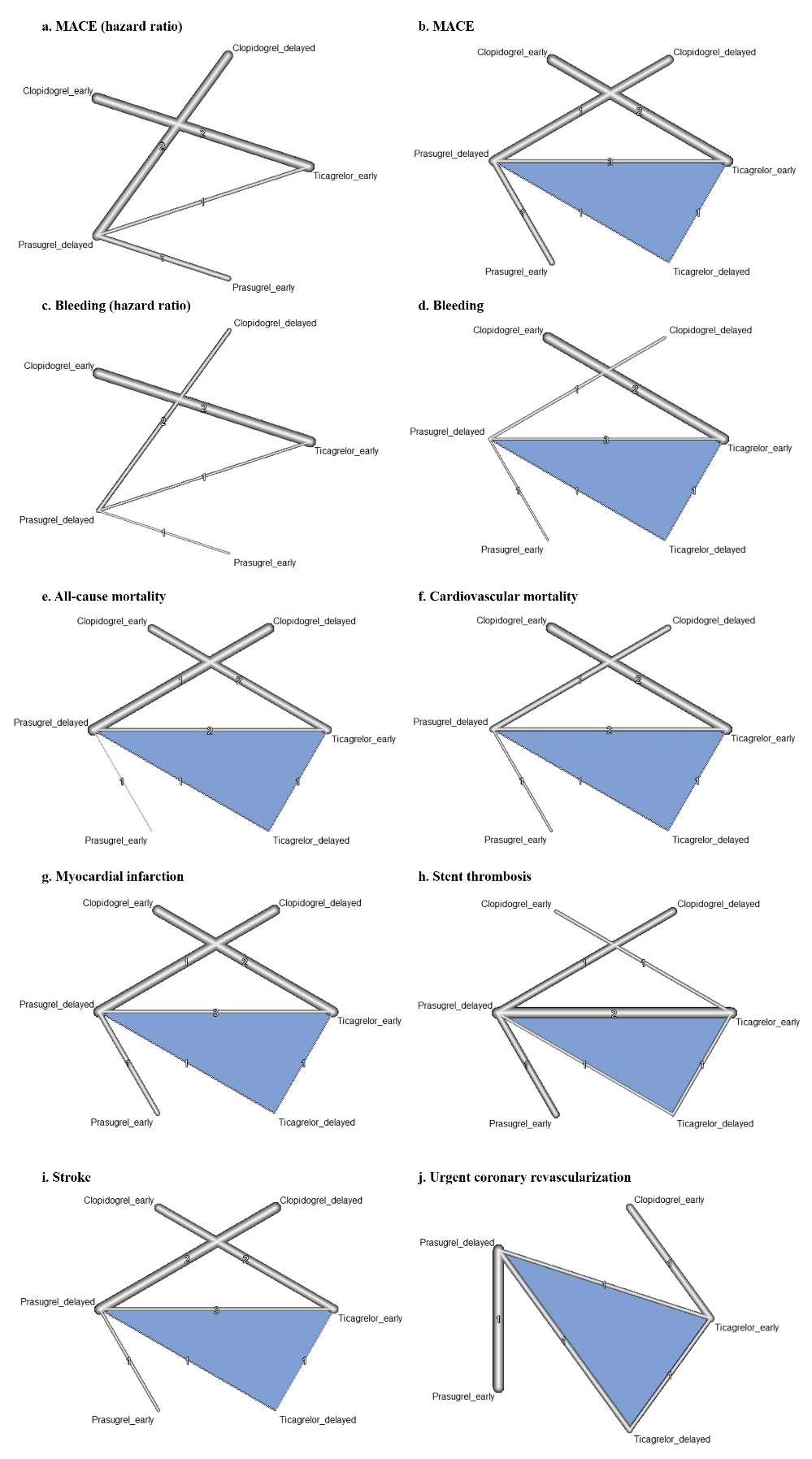
**

**Figure S3. Effect of P2Y12 inhibitors on primary and secondary outcomes in comparison to early-treatment with clopidogrel.**

**
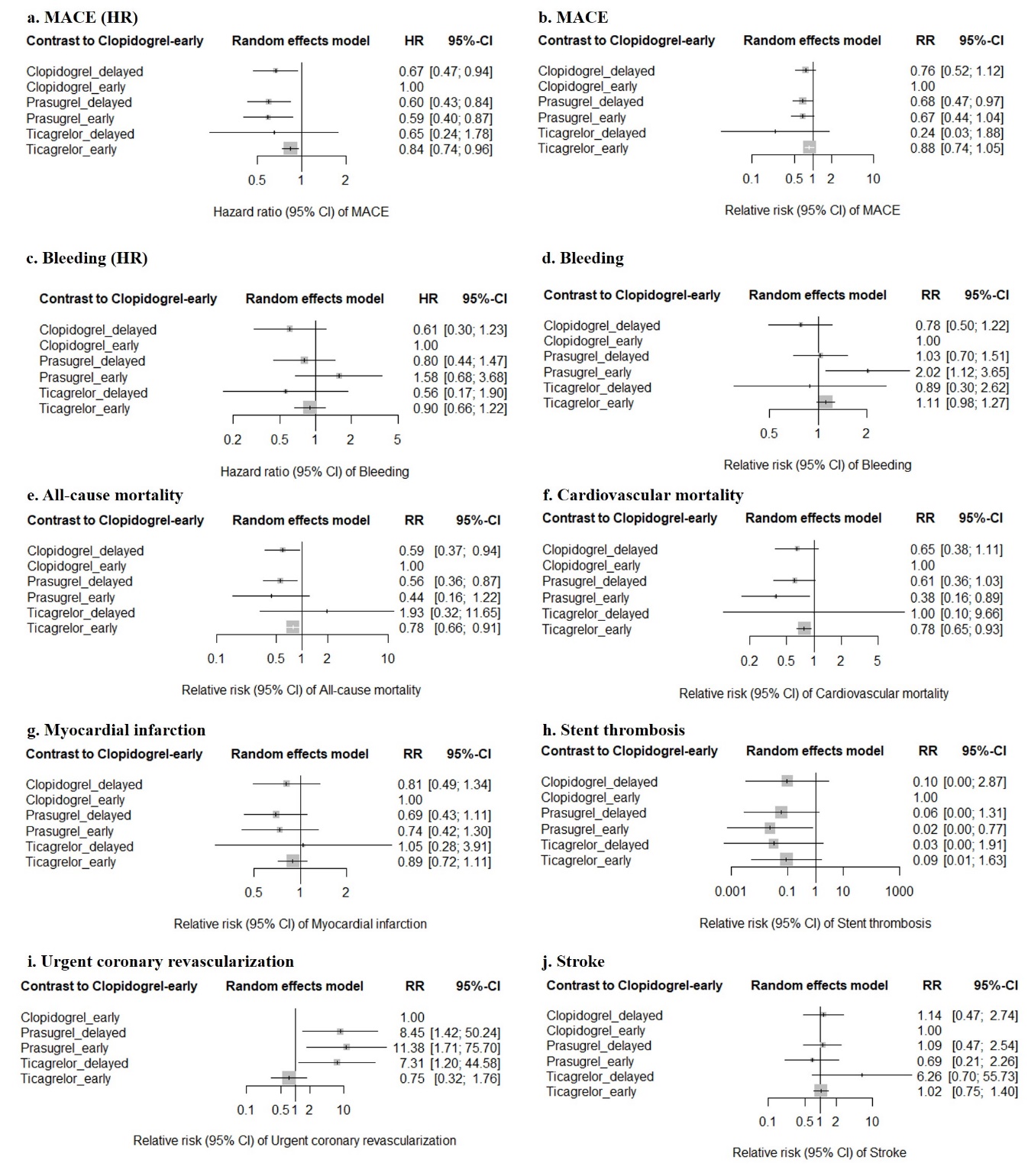
**

MACE, major adverse cardiovascular events; RR, risk ratio; CI, confidence interval; HR, hazard ratio.

**Figure S4. Direct and indirect effects of P2Y12 inhibitors on MACE (HR).**

**
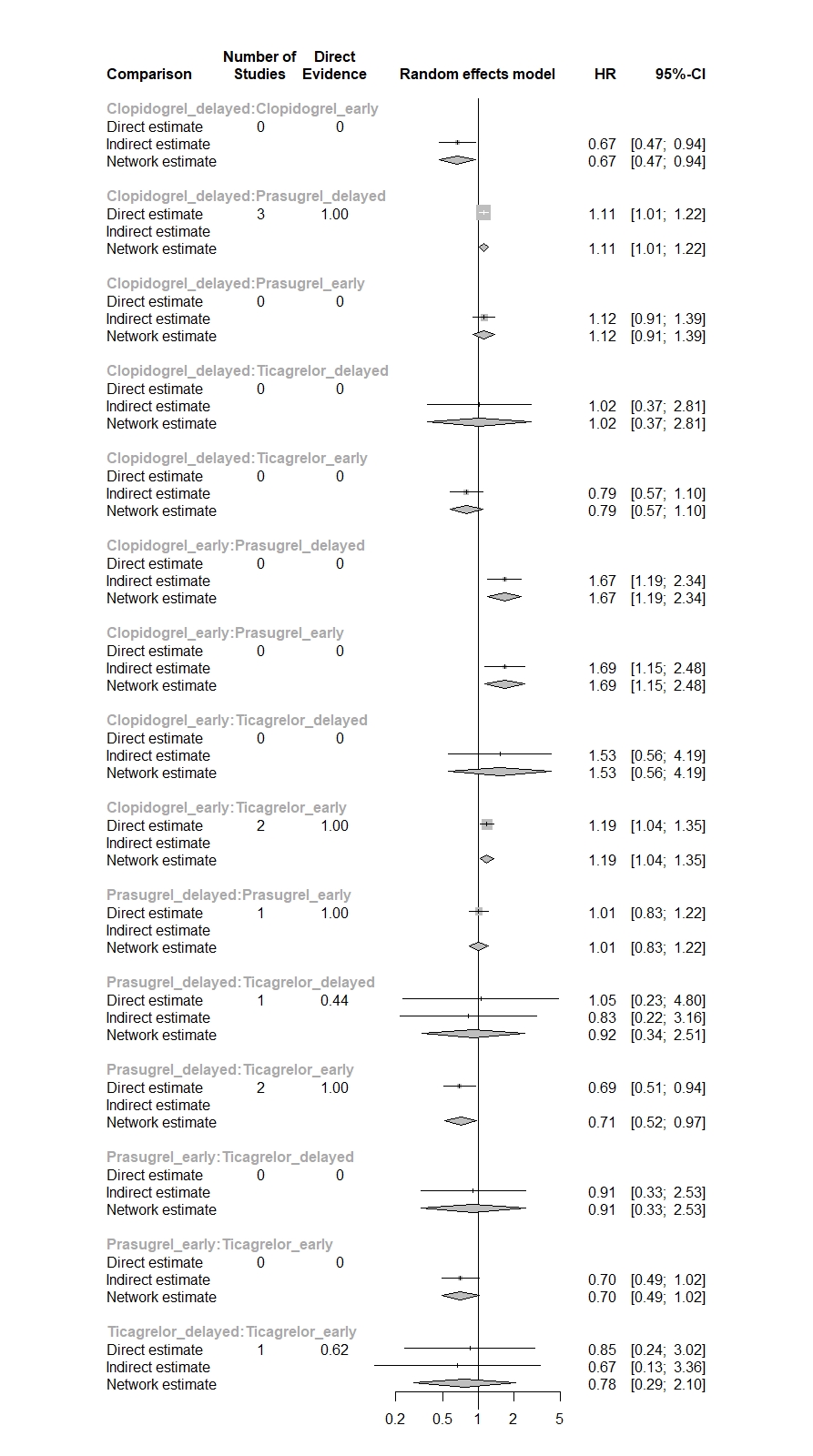
**

MACE, major adverse cardiovascular events; HR, hazard ratio; CI, confidence interval.

**Figure S5. Direct and indirect effects of P2Y12 inhibitors on MACE.**

**
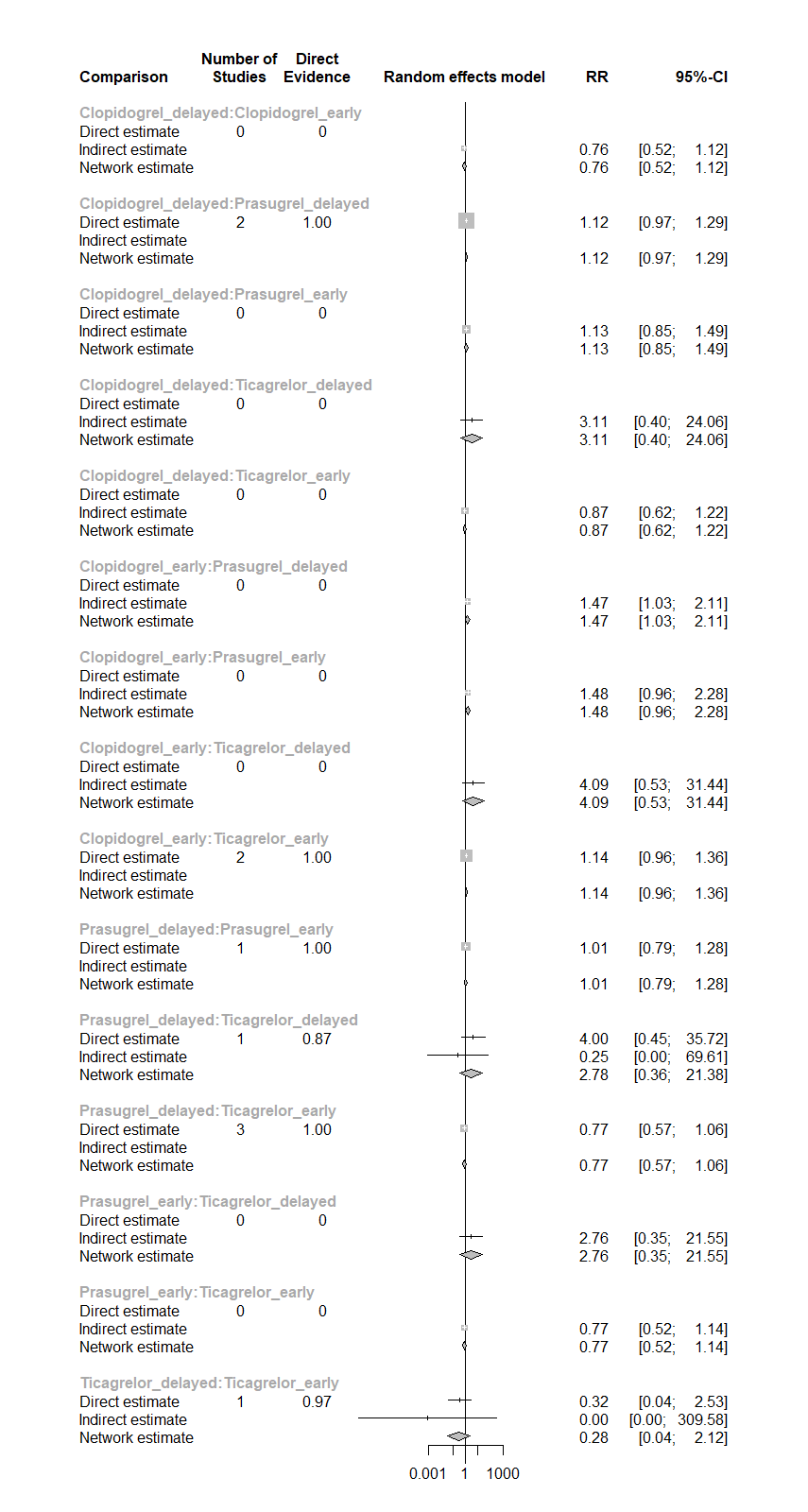
**

MACE, major adverse cardiovascular events; RR, risk ratio; CI, confidence interval.

**Figure S6. Direct and indirect effects of P2Y12 inhibitors on bleeding (HR).**

**
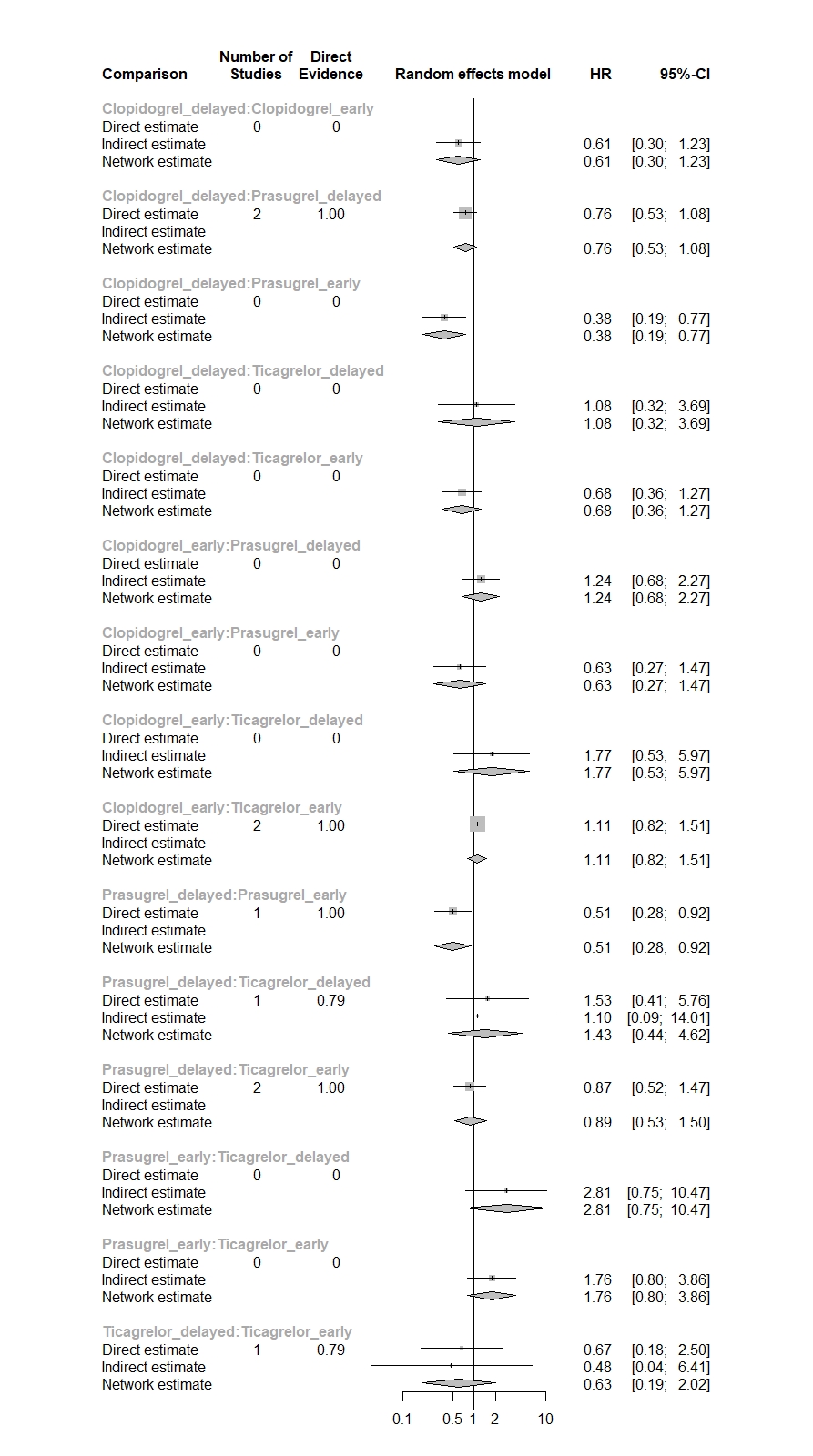
**

HR, risk ratio; CI, confidence interval.

**Figure S7. Direct and indirect effects of P2Y12 inhibitors on bleeding.**

**
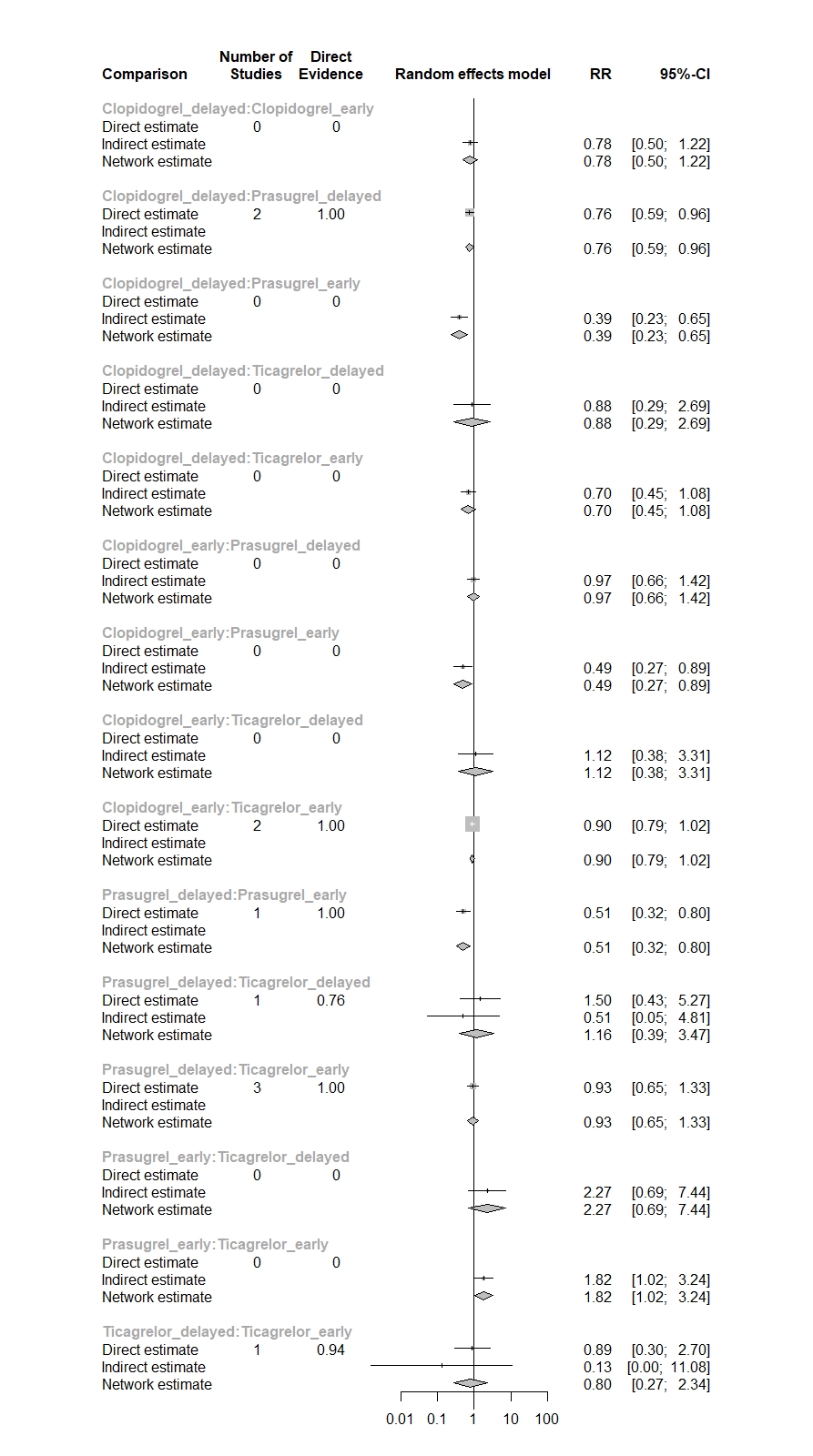
**

RR, risk ratio; CI, confidence interval.

**Figure S8. Direct and indirect effects of P2Y12 inhibitors on all-cause mortality.**

**
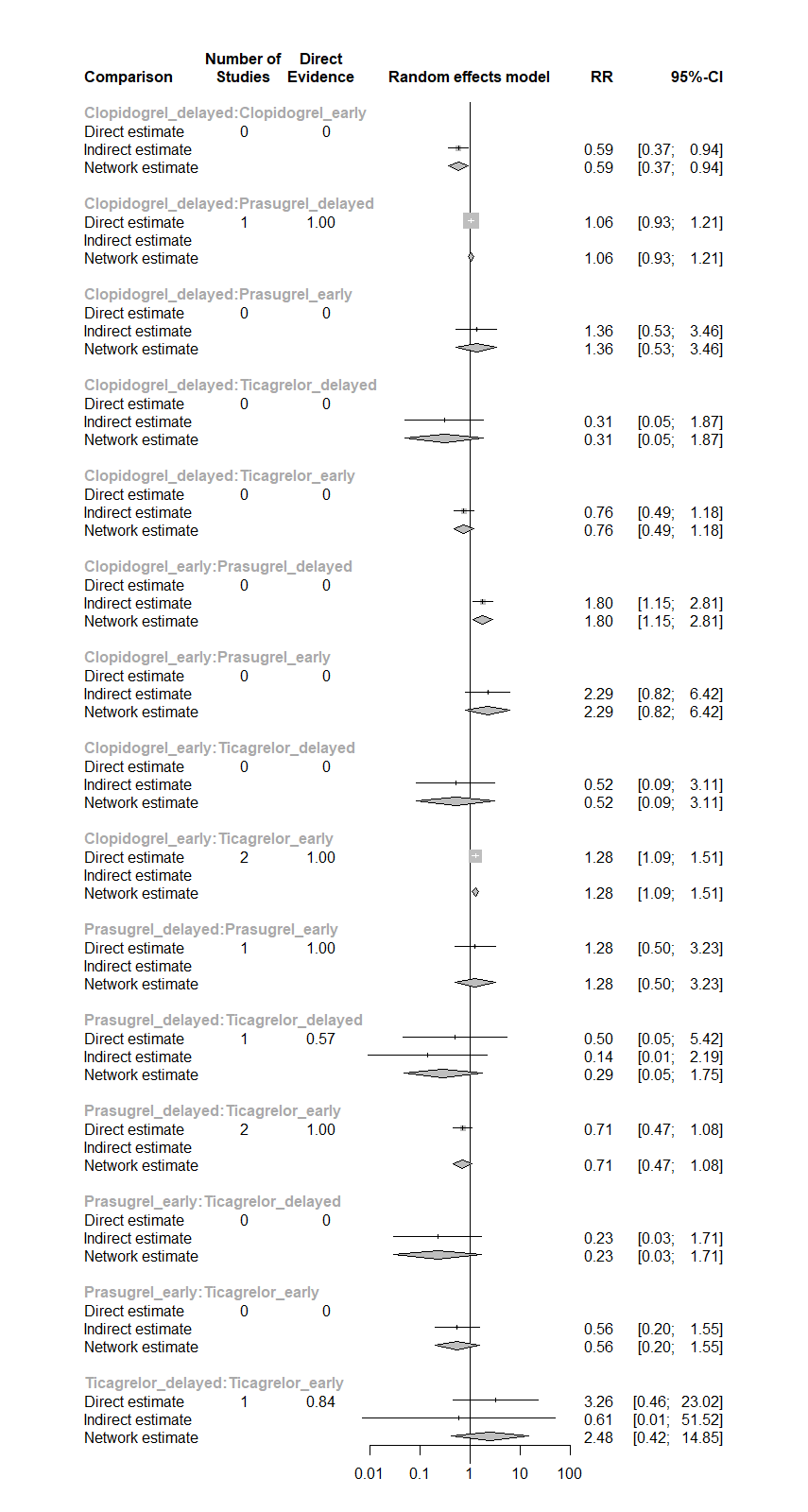
**

RR, risk ratio; CI, confidence interval.

**Figure S9. Direct and indirect effects of P2Y12 inhibitors on cardiovascular mortality.**

**
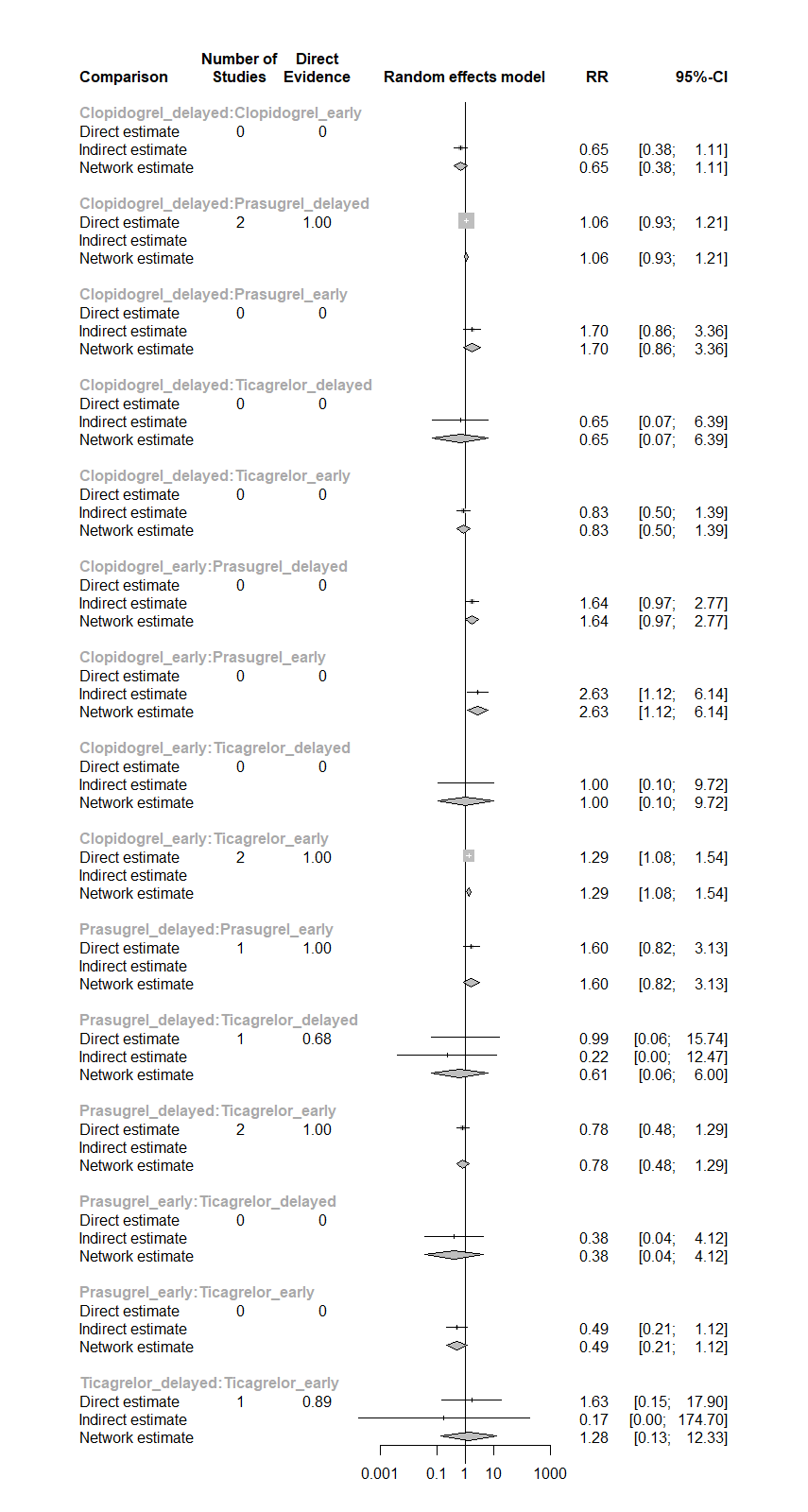
**

RR, risk ratio; CI, confidence interval.

**Figure S10. Direct and indirect effects of P2Y12 inhibitors on myocardial infarction.**

**
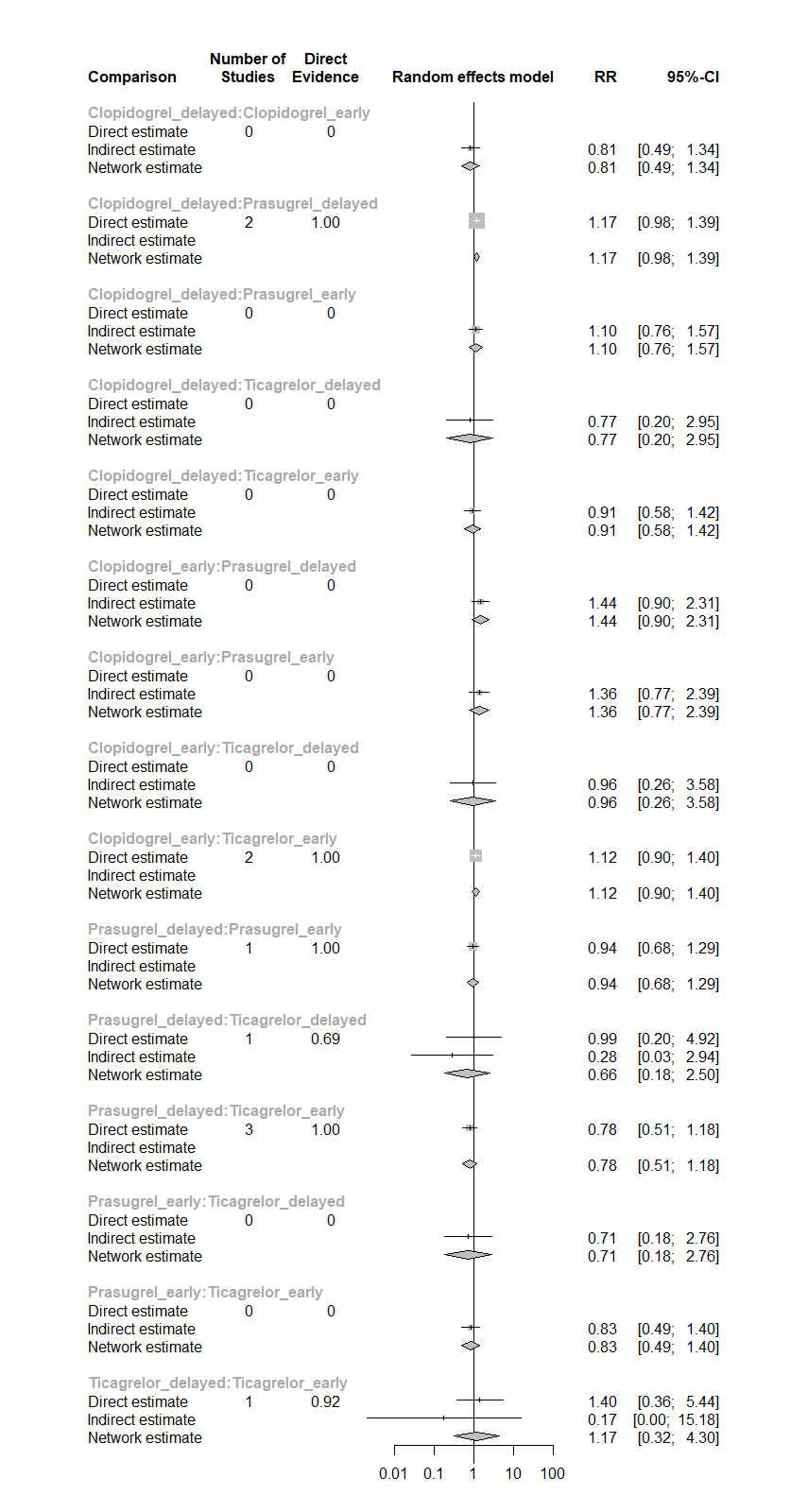
**

RR, risk ratio; CI, confidence interval.

**Figure S11. Direct and indirect effects of P2Y12 inhibitors on stent thrombosis.**

**
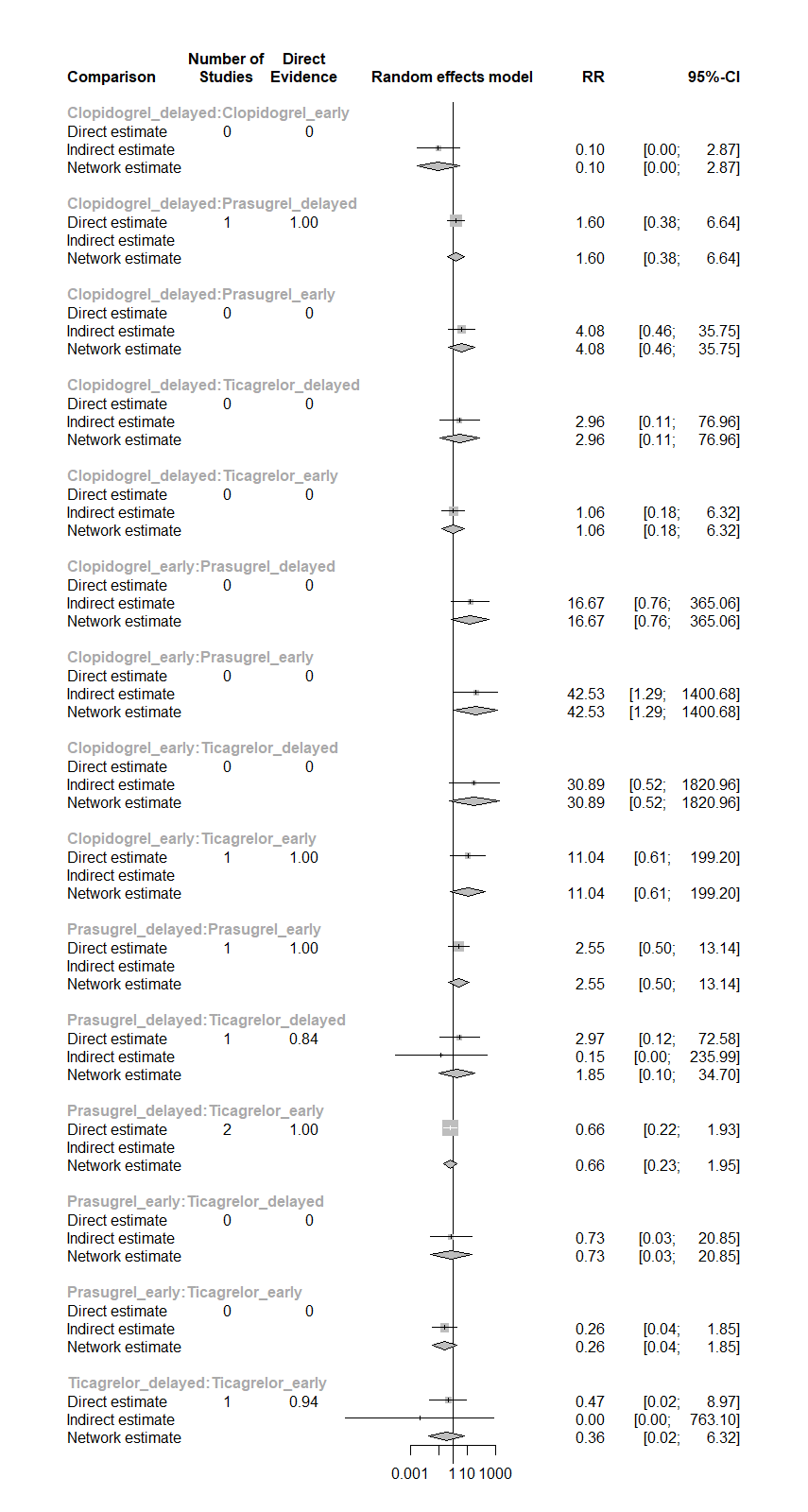
**

RR, risk ratio; CI, confidence interval.

**Figure S12. Direct and indirect effects of P2Y12 inhibitors on stroke.**

**
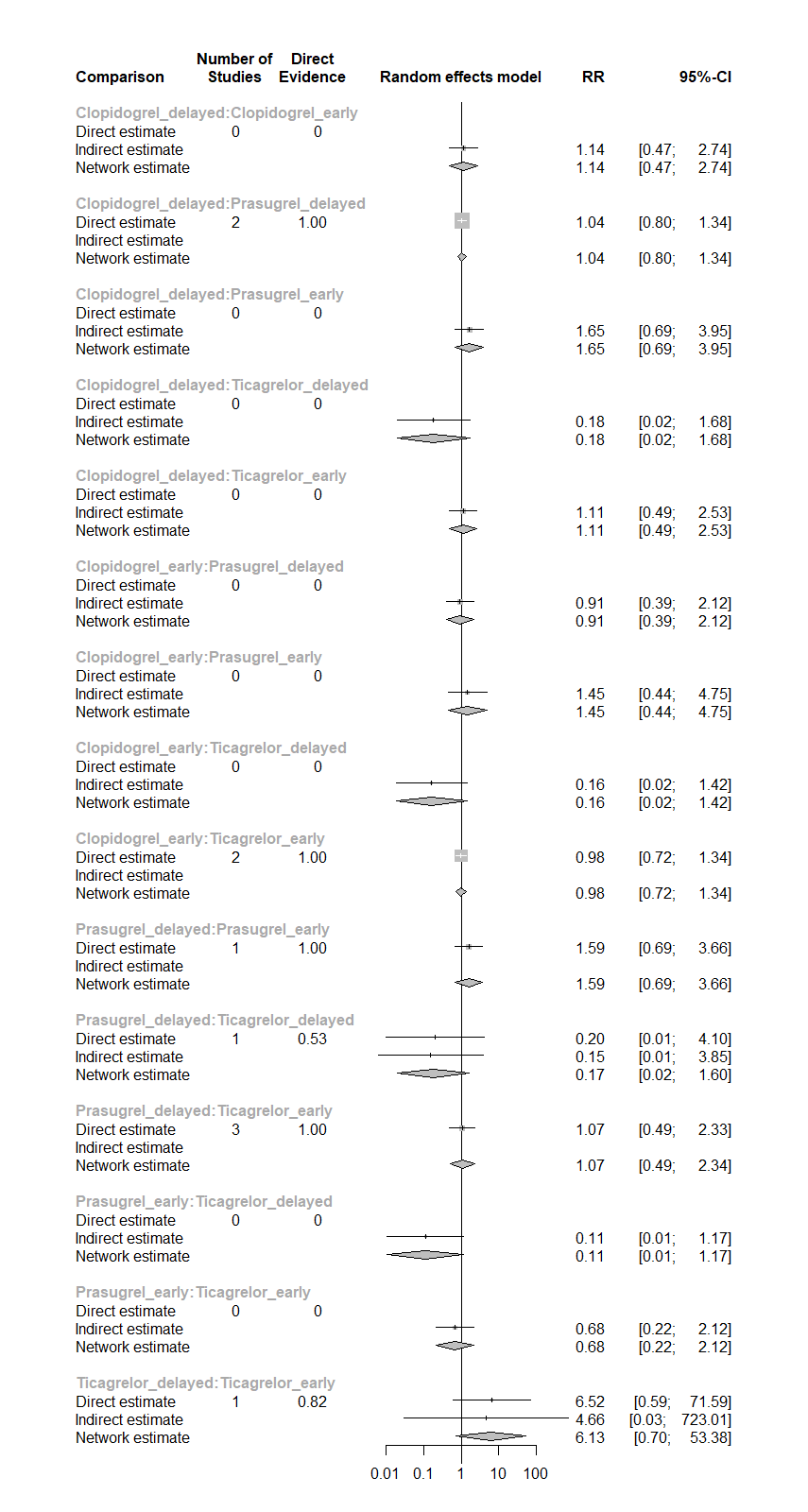
**

RR, risk ratio; CI, confidence interval.

**Figure S13. Direct and indirect effects of P2Y12 inhibitors on urgent coronary revascularization.**

**
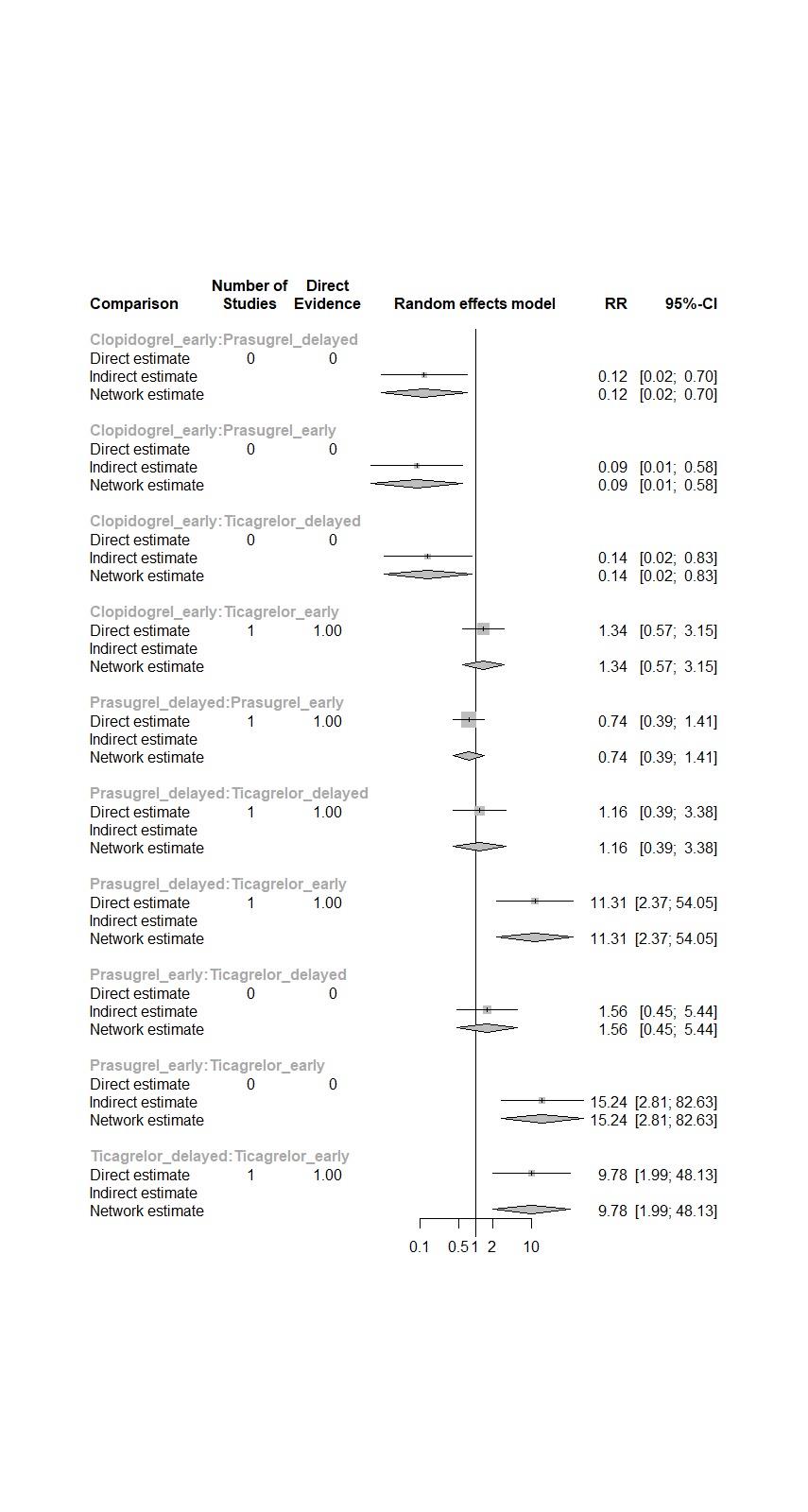
**

RR, risk ratio; CI, confidence interval.
